# Pre-Columbian zoonotic enteric parasites: An insight into Puerto Rican indigenous culture diets and life styles

**DOI:** 10.1371/journal.pone.0227810

**Published:** 2020-01-30

**Authors:** Rosana Wiscovitch-Russo, Jessica Rivera-Perez, Yvonne M. Narganes-Storde, Erileen García-Roldán, Lucy Bunkley-Williams, Raul Cano, Gary A. Toranzos

**Affiliations:** 1 Environmental Microbiology Laboratory, University of Puerto Rico, San Juan, Puerto Rico; 2 Ecosystems & Global Change, Manaaki Whenua-Landcare Research, Lincoln, New Zealand; 3 Center for Archaeological Research, University of Puerto Rico, San Juan, Puerto Rico; 4 Department of Biology, University of Puerto Rico, Mayagüez, Puerto Rico; 5 The BioCollective, Denver, Colorado, United States of America; Seoul National University College of Medicine, REPUBLIC OF KOREA

## Abstract

The pre-Columbian Huecoid and Saladoid cultures were agricultural ethnic groups that supplemented their diets by fishing, hunting and scavenging. Archaeological deposits associated to these cultures contained a variety of faunal osseous remains that hinted at the cultures’ diets. The present study identified zoonotic parasites that may have infected these two cultures as a result of their diets. We used metagenomic sequencing and microscopy data from 540–1,400 year old coprolites as well as the zooarchaeological data to recreate the possible interactions between zoonotic parasites and their hosts. Microscopy revealed *Diphyllobothrium* spp. and *Dipylidium caninum* eggs along with unidentified cestode and trematode eggs. DNA sequencing together with functional prediction and phylogenetic inference identified reads of *Cryptosporidium* spp., *Giardia intestinalis* and *Schistosoma* spp. The complimentary nature of the molecular, microscopy and zooarchaeology data provided additional insight into the detected zoonotic parasites’ potential host range. Network modeling revealed that rodents and canids living in close proximity to these cultures were most likely the main source of these zoonotic parasite infections.

## Author summary

This communication includes a descriptive analysis of zoonotic parasites infecting Caribbean pre-Columbian cultures in Vieques and network modeling of parasite-host interaction. Our conclusions were based on microscopy and DNA reads extracted from approximately 1,400 to 540 year-old coprolites (un-calibrated years before present). Age and exposure to taphonomic conditions most likely caused the degradation of a portion of the organic material preserved in the coprolites, including parasite eggs, cysts and genetic material. In this study, singleton parasite reads were detected in the metagenomic data. This was likely the outcome of various factors. For instance, non-targeted metagenomic sequencing mainly obtains reads from abundant organisms within the sample, and in the case of fecal metagenomic datasets, these are commonly overpowered by high numbers of bacterial cells. In addition to small amounts of detectable parasite DNA in the feces, ancient DNA damage causes difficulty during sequencing and subsequent alignment of reads to an extant reference sequence. This along with limited representation of parasite genomes in curated reference databases limits the prediction of parasite homologue reads [1]. Furthermore, although contamination and sequencing errors are also a possibility, we believe that this is not the case as precautions were taken to avoid contamination with extant DNA. To overcome these challenges, we performed phylogenetic inference of predicted amino acid homologues to obtain as close as possible to an accurate taxonomic identification. To complement the inherent limitations of the analyses, some of the conclusions presented in this article include intellectual assumptions by the authors based on established knowledge of the field after analyzing and discussing the data.

## Introduction

**Fig 1:**
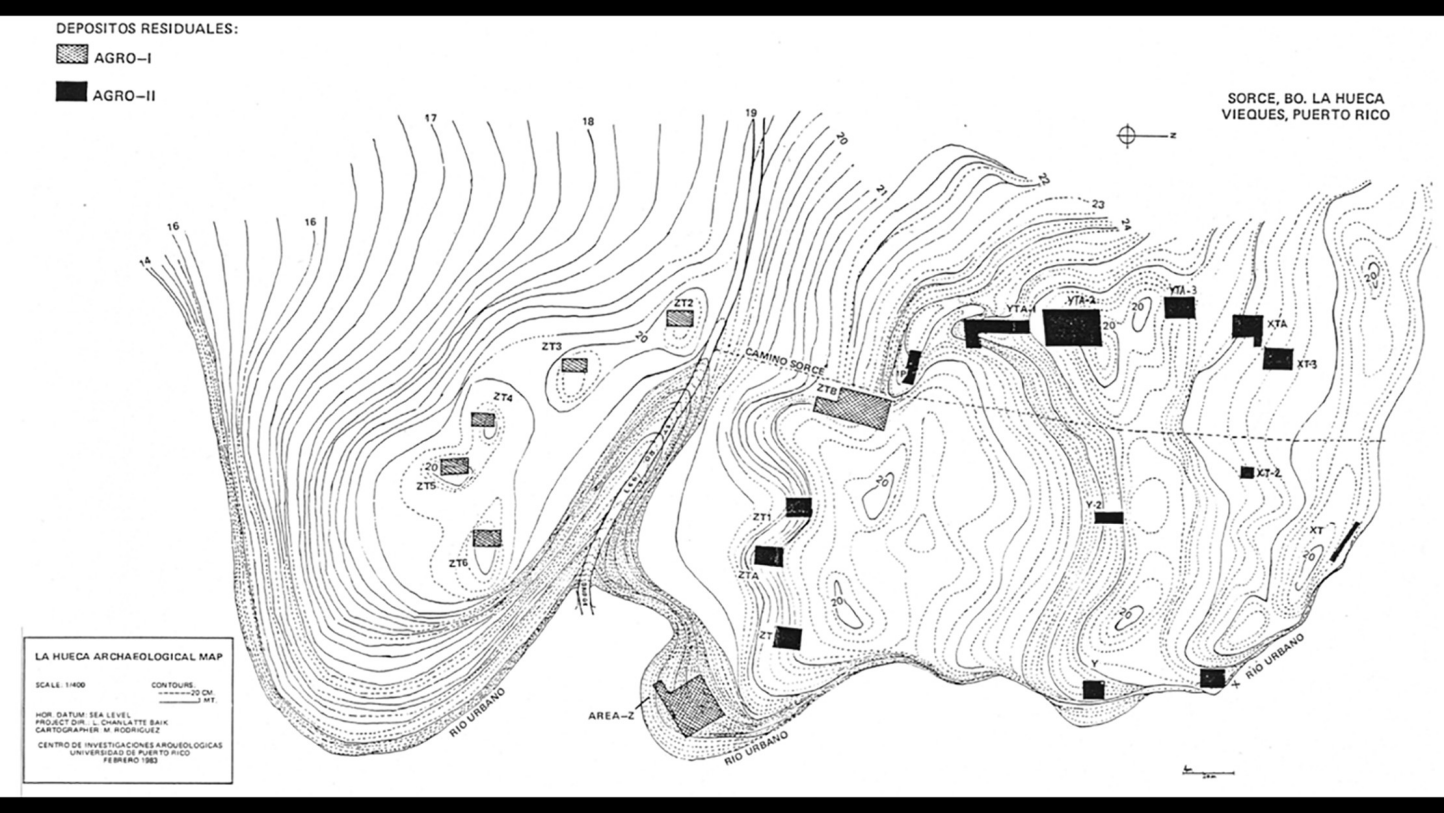
Topological map of the Sorcé settlement demonstrating the Huecoid (gray) and Saladoid (black) deposits. Each archaeological deposit was separated by a distance of 5–150 meters. Faunal osseous remains were retrieved from Z and YTA-2 deposits. The coprolites were retrieved from Z, ZT, YTA-1 and YTA-2 deposits. A stream is seen at the edge of the topology map, labeled as “Rio Urbano”. Reprinted from [2] under a CC BY license, with permission from [Museum of History, Anthropology and Art, University of Puerto Rico], original copyright [2005].

The Huecoid and Saladoid cultures co-existed at the Sorcé Site in Vieques, Puerto Rico over 1,400 years ago. These cultures originated in South America and migrated to the Caribbean Antilles in separate migratory waves [2]. Despite migrating to the Antilles, the Huecoid and Saladoid maintained their ancestral heritage, as evidenced by the unique pottery and semi-precious stone work [2]. These archaeological artifacts distinguished the cultures origins from the Andean region presumably from present day Bolivia and Peru (Huecoid) and present day Venezuela (Saladoid) respectively [2]. The archaeological deposits (described as dumpsites) contained shattered pottery, faunal remains, lithic and shell tools as well as the coprolites analyzed in this study [2–3]. In the Sorcé settlement Huecoid and Saladoid housing was established on a high plain overlooking the Caribbean Sea. The settlement was composed of three ascending levels where the individuals built their lodges and used the slope to discard their waste causing an accumulation of these items over time (Fig 1). A nearby creek slope was also used as a dumpsite, resulting in sites rich in archaeological artifacts. The sites were culturally distinguishable based on the characteristic artifacts present at each site. For instance, the Huecoid deposits were characterized by plain pottery and an abundance of elaborately carved semi-precious stones whereas the Saladoid deposits were characterized by red and white painted pottery and a profusion of carved shell ornaments. Therefore, the excavated osseous remains and coprolites were categorized by culture according to this criterion.

These groups supplemented their diets by fishing and hunting as well as gathering invertebrates such as mollusks and crustaceans [4]. Osseous remains of a variety of birds, reptiles, fishes, and mammals (including rodents, canids, and other organisms) were obtained from the archaeological deposits [3–4]. The identified osseous remains were likely part of their diets as a protein source, with the exception of canids that may have been an occasional food source [3–4]. Faunal osseous remains provided an opportunity to infer possible zoonotic parasite transmission, as the coexistence and consumption of these animals possibly made the cultures susceptible to zoonotic infections.

The Huecoids and Saladoids co-existed in the settlement. The dwellings were separated by a short distance (15–150 meters) [3,5]. According to Barret *et al*. [6], small and diffuse groups of individuals could not support a large number of infectious agents. In theory, parasite diversity increased with the establishment of permanent settlements, that included the domestication of animals and an increase in agricultural practices facilitating transmission of both anthroponotic and zoonotic parasites [6–9]. Based on this knowledge, we suspect that zoonotic and human-specific parasites were potentially sustainable in the settlement. As with other cultures [10–11], the Huecoids and Saladoids were susceptible to zoonotic infections brought about by interacting with infected animals or consuming the parasite-infected host.

The present study combined Next-Generation Sequencing data with microscopy and zooarchaeological data to elucidate the interactions between zoonotic parasites and hosts in the Sorcé settlement. Using microscopy and shotgun metagenomics, parasite eggs and DNA were detected revealing zoonotic infections present in the inhabitants. Zooarchaeological data supplemented evidence of potential animal hosts and modes of transmission of the detected zoonotic parasites. The data was examined using network modeling to interpret zoonotic parasite-host interactions.

## Methods

### Archaeological analyses

Excavations at the Sorcé site (18˚05’ 56” Latitude North and 65˚29’ 34” Longitude West) in the Island of Vieques, Puerto Rico from 1977 to 1984 recovered faunal osseous remains from deposits Z and YTA-2, while the coprolites were recovered from deposits Z, ZT, YTA-1 and YTA-2 (Fig 1). No permits were required, the excavations took place in a private property (Sorcé, Vieques) and the owner’s consent was provided for the excavations by the Archaeological Research Center at the University of Puerto Rico, which complied with all relevant regulations. Coprolites ages (Table 1) were determined by radiocarbon dating of material associated with the samples [2]. Faunal osseous remains were identified by Narganes-Storde [3] via comparative analysis with a synoptic collection from the Zooarchaeology Laboratory of the Florida State Museum and the Center for Archaeological Research of the University of Puerto Rico (S2 Table). The coprolites and faunal osseous remains belong to an archaeological collection and they are permanently deposited in the Center for Archaeological Research of the University of Puerto Rico.

**Table 1:** Description of the coprolites used in the molecular analysis.

Registration	Culture	Deposit	Quadrant	Depth	Radiocarbon Date
**5.2003.0002**	Huecoid	Z	Z—37	0.20–0.40 cm.	470 A.D.
**5.2003.0006**	Huecoid	Z	Z—L	0.70 cm.	Circa 385 A.D.
**5.2003.0007**	Huecoid	Z	Z—M	1.20 mt.	Circa 450 A.D.
**5.2003.0010**	Huecoid	Z	Z—W	1.80 mt.	Circa 245 A.D.
**5.2003.0011**	Huecoid	Z	Z—W	2.00 mt.	215–220 A.D.
**5.2003.0012**	Huecoid	Z	Z—X	0.60 cm.	470–600 A.D.
**5.2003.0014**	Saladoid	YTA-2	J—22	0.80 cm.	270–385 A.D.
**5.2003.0015**	Saladoid	YTA-2	H—21	1.20 mt.	230–385 A.D.
**5.2003.0016**	Saladoid	YTA-2	M—25	0.40 cm.	230–385 A.D.
**5.2003.0019**	Saladoid	YTA-1	I—5	0.60 cm.	335–395 A.D.

### Microscopy

Portions of the coprolites (n = 20) were processed for microscopy using traditional flotation methods which allowed for the systematic observation of helminth eggs (S1 Table) [12]. Briefly, one gram of each sample was rehydrated in 14ml of 0.5% trisodium phosphate for 72 hours [13], shaken vigorously and filtered (1500μm mesh) to eliminate debris. Subsequently, 1ml of 10% acetic formalin solution was added per 10g of filtrate [14]. The samples were allowed to settle for 72 hours [15], and ten microscope slides were prepared using 50μl of sediment mixed with a drop of glycerin. Each slide was covered with a 20x20 cover slip and scanned microscopically in a serpentine fashion [16].

### Preparation, DNA extraction and sequencing

Extraction and sequencing of ancient DNA (aDNA) was performed as described by Rivera-Perez *et al*. [17]. Briefly, nine coprolites, namely Huecoid (n = 5) and Saladoid (n = 4) were selected for shotgun metagenomic sequencing. Coprolites were processed in a reserved area of the laboratory for ancient DNA to avoid contamination. The coprolites were processed separately in a class II biosafety cabinet that was routinely disinfected with 70% ethanol and exposed to UV light for at least 30 minutes before and after use. All instruments were autoclaved and baked overnight at >100°C to denature any extraneous DNA. To reduce the presence of soil microbiota, the exterior layer was removed and the core of each coprolite was used. The cores were grounded separately using a sterile mortar and pestle. Ancient DNA was isolated using a PowerSoil DNA Extraction Kit (Mo Bio Laboratories, Carlsbad, CA) according to manufacturer’s instructions. All samples were hydrated overnight in sterile C1 buffer at 4˚C prior to extraction. Using standard glycogen precipitation, 10ul of aDNA were pooled according to ethnic group (MixS1 and MixH1) to compensate for low concentrations of aDNA. The concentration of aDNA was assessed using a Qubit® dsDNA HS Assay Kit (Life Technologies) and sequenced at a commercial facility (MR DNA Research lab in Shallowater, TX). REPLI-g Midi kit (Qiagen) was used for non-targeted whole genome amplification (WGA) followed by Nextera library preparation kit and sequencing with Illumina MiSeq system.

### Putative parasite sequences and phylogenetic assignment

Fastq files produced by Illumina MiSeq were assessed through MG-RAST [18] for quality control and read length exclusion based on default parameters. Amino acid predictions of the metagenomic datasets were conducted using BLASTX [19] against a non-redundant protein NCBI database (National Center for Biotechnology Information). To address the damage present in aDNA [20], the cut-off value for functional identification was set at an E-value of <-15. Sequences with specific functions and high alignment scores to parasite reference reads were verified using MEGA 7 [21] for accurate taxonomic identification of BLASTX homologous results. BLAST hits were downloaded from NCBI and concatenated into fasta files with the putative parasite read extracted from the metagenomic dataset. The fasta file were imported to MEGA 7 for multiple sequence alignment using MUSCLE [22]. Substitution model was selected using Find Best DNA/Protein Models. The suggested model was then used to create a pairwise distance matrix and construct maximum likelihood phylogenetic tree using 1000 bootstrap iterations (see Supplementary Material).

### Modeling parasite-host interaction

HelminthR [23] and rglobi (global biotic interactions) [24] curated databases for host-parasite interactions were used to verify zooarchaeological data as potential hosts for the identified parasites. Parasite-host interactions were reconstructed by generating a directional dataset of the identified parasites (detected by microscopy or molecular analysis) and general descriptions of potential host detected from the zooarchaeological data. Parasite-host interactions were modeled using the network graphical R package ‘igraph’ [25].

### Supplementary materials

Parasite sequences are available in S1 Data. HelminthR and rglobi search results are available in S2 & S3 Data. The generated dataset and Rscript used in this study are included in S4 & S5 Data. BLAST homology of putative reads of the consumed animal are included in S6 & S7 Data.

## Results

Microscopy analysis revealed *Diphyllobothrium* spp. and *Dipylidium caninum* eggs along with unidentified cestode and trematode eggs. One unidentified cestode egg was presumed to be of a hymenolepidid tapeworm (Table 2[12]. Parasite host ranges differ between species, therefore parasite DNA would need to be identified to the species level to determine its specific host range; this would require strict alignment parameters (E-value 0.0 and Percentage of Identity >98%). However, it is unlikely to achieve high alignment scores with highly degraded aDNA [26]. In each DNA alignment, statistical evaluation with phylogenetic associations was carried out to eliminate potential false assignments of BLAST hits and obtain an accurate taxonomic identification [27–29]. BLASTX predictions identified putative reads of important parasites of animal hosts, such as *Cryptosporidium* spp., *Eimeria necatrix*, *Giardia intestinalis*, *Perkinsus marinus*, *Toxoplasma gondii*, *Hymenolepis microstoma*, and *Schistosoma mansoni*. After BLASTX prediction, one read produced sole homology to a glutamate dehydrogenase of *Giardia intestinalis* (disambiguous), thus there was no need for subsequent phylogenetic analysis (S7 Table). *Cryptosporidium* spp. and *Schistosoma* spp. reads were confirmed by phylogenetic analysis. However, phylogenetic inference excluded *H*. *microstoma* and *T*. *gondii* as potential zoonotic infections, as their corresponding reads resulted in a best match to Ascomycota (S11 and S12 Figs). BLASTX prediction for *E*. *necatrix* and *P*. *marinus* produced inconsistent results in pairwise distance matrix and maximum likelihood phylogenetic inference, and were therefore excluded from the network modeling.

**Table 2:** Potential zoonotic parasites identified by microscopy and sequence from the metagenomic datasets. The amount of unidentified cestode eggs detected in the Huecoid (n = 111) and Saladoid coprolites (n = 147), respectively. A total of 26 unidentified trematode eggs were detected in the Saladoid coprolites via microscopy. Microscopy images of parasite eggs are available as supplementary material (S13 Fig).

Predicted Parasite Genera	Total Reads		Eggs Detected by Microscopy
	Huecoid	Saladoid	
*Cryptosporidium*	**-**	**1**	ND
*Giardia intestinalis*	**-**	**1**	ND
*Diphyllobothrium*	**-**	**-**	A total of 26 eggs were detected in one Saladoid coprolite.
*Dipylidium caninum*	**-**	**-**	A total of 30 eggs were detected in one Saladoid coprolite.
*Hymenolepis*	**-**	**-**	One cestode egg presumed to be a Hymenolepid.
*Schistosoma*	**-**	**2**	ND

*ND = None Detected

**Fig 2:**
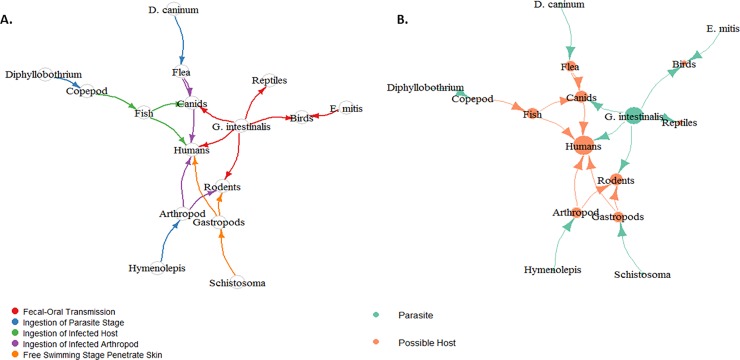
Directional network modeling: (A) edges describing the parasite-host transmission and (B) nodes size reflecting the Eigen vector centrality (refer to S3 Table for Eigen values). The model reflects the relationship types as edges (arrows) and the subjects as nodes (circles). Network modeling evaluates the relationship or interaction between the parasite and the host. Eigen vector centrality is reflected by the node size and the Eigen value measures the influence of a node in the network. A high Eigen value means that a node is connected to many nodes which themselves have a high scores.

Network modeling was used to recreate parasite-host interactions. Eigen values and degrees of connectivity were used to measure the importance of a node in the network. The human node was assigned a higher degree of connectivity (n = 5) and Eigen value (n = 1.00) (Fig 2B and S3 Table), reflecting the modeling of zoonotic infection and the use of human coprolites. This was further supported by the identification of human-specific parasites by microscopy, including *Ascaris lumbricoides*, Ancylostomatidae and *Trichuris trichiura* [12]. Ancyclostomatidae was later identified as *Necator americanus* by molecular analysis (S17 Table and S7 Fig). The second highest Eigen value (n = 0.89) was assigned to *G*. *intestinalis* (Fig 2B and S3 Table), the parasite has a wide host range in the network directly infecting humans, rodents, canids and reptiles via fecal oral transmission (Fig 2A and Table 3). The third highest degree of connectivity (n = 3) and Eigen value (n = 0.64) was assigned to rodents and canids (Fig 2B and S3 Table), reflecting a crucial role of these hosts in the transmission of zoonotic parasites in the settlement. Furthermore, the modeled network has several nodes that are highly connected and representative of a real network (S1 Fig).

**Table 3:** General information of parasites detected in Huecoid and Saladoid cultures. The table supplements and summarizes the information depicted in Fig 2A.

Description: Related to Human Infection	Ingestion of contaminated food or water source, including infection through fomites.	There is no evidence of *Eimeria* being hazards to humans.	Ingestion of contaminated food or water source, including infection through fomites.	There is no evidence of *P*. *marinus being* hazards to humans.	Ingestion of raw or under cooked infected fish.	Human are accidental host acquire the parasitosis by ingesting the flea vector.	Ingestion of cysticercoid-infected arthropod.	Infective cercariae swims and penetrates the skin of the human host.
**Transmission**	Fecal-Oral Transmission	Fecal-Oral Transmission	Fecal-Oral Transmission	Diffusion of Parasite in Water	Ingestion of Infected Secondary Intermediate Host	Ingestion of Infected Arthropod	Ingestion of Infected Arthropod	Free Swimming Cercariae Penetrate Skin
**Second Intermediate**	-	-	-	-	Fish	-	-	-
**First Intermediate**	-	-	-	-	Copepods	Flea Vector: *Ctenocephalides spp*.	Arthropods	Gastropods
**Definitive**	-	Birds	Wide Host Range	Bivalves: *Crassostrea* or *Mya*	Mammals	Canids	Mammals	Mammals
**Parasites Detected**	*Cryptosporidium*	*Eimeria*	*Giardia intestinalis*	*Perkinsus marinus*	*Diphyllobothrium*	*Dipylidium caninum*	*Hymenolepid*	*Schistosoma*

## Discussion

Results of ancient DNA and microscopy analyses of Huecoid and Saladoid coprolites were inconsistent depending on the method used (Table 2). Firstly, protozoa detected via sequencing and phylogenetic inference were not detected by microscopy. It is possible that the composition of protozoan cyst may undergo morphological changes as a result of the taphonomic processes that preserved these coprolites, and the small size (4–12 micrometers) makes them difficult to identify using light microscopy [30]. Previously protozoan cysts were successfully detected in ancient biological artifacts by immunofluorescence [1]. It is clear that different cysts may be refractory to degradation for different lengths of time and likely the resistance of the cyst walls determines the protection of the nucleic acids. Second, *Diphyllobothrium*, *D*. *caninum* and presumed hymenolepidid eggs were identified by microscopy [12], but no DNA sequences were assigned homology to these tapeworms. However, this tendency was observed before by Côte *et al*. [31]. When comparing microscopy to PCR-based genotyping of human gastrointestinal parasites Côte *et al*. [31] observed a high proportion of helminth eggs but DNA sequences were not detected for *Trichuris* spp., *Ascaris* spp. nor *Taenia* spp. These observations could have been due to degradation of the genetic material, primer-based variability if the eggs belonged to animal-borne parasites or even to larvae exiting the egg after defecation [31]. Although in our analysis we did not use conserved primers, arguably, the absence of parasite DNA accompanied with the positive identification of the eggs in our data could have resulted from degradation of genetic material or a reflection of the limited variance of helminth genomes in curated reference databases [1].

Network modeling is a flexible and useful manner to represent the subjects of a dataset and their relationships. Network analysis also allows for further interpretation on how diets and life styles influence the transmission of zoonotic infections. In this study, network modeling was used to recreate parasite-host interactions in Sorcé. The subjects in the network were the identified parasites (through microscopy or phylogenetically inferred sequences) and the potential hosts of the pre-Columbian settlements (as evidenced by the osseous remains). Although small organisms with fragile exoskeletons were not found in the zooarchaeological data, they were included in the network. This exception was made for copepods and arthropods (such as beetles and fleas) since they are ubiquitous in the environment and are essential for the development of some parasites at certain stages of their life cycles. For instance, *Diphyllobothrium*, *D*. *caninum*, and *Hymenolepis* spp. rely on intermediate hosts with fragile exoskeletons to complete their life-cycles. Overall, the detected parasites did not share a similar host range (Table 3), each parasite requires a specific host and mode of transmission to achieve its full development (Fig 2A).

### Fecal-oral transmission

*Giardia intestinalis* is a zoonotic protozoan parasite with a wide host range transmitted by the fecal-oral route [32]. *Cryptosporidium* spp. and *Giardia* spp. life cycles are direct and develop to completion within one host [32–33]. Ortega and Bonavia [30] detected *Giardia* spp. and *Cryptosporidium* spp. in pre-Columbian Peruvian coprolites, although co-infection was not observed in the samples. *Giardia* spp. and *Cryptosporidium* spp. were identified in our metagenomic datasets. However, *Cryptosporidium* was not identified to the species level and therefore was not included in the network analysis as each of its species has a varied host range [32]. In our study *G*. *intestinalis* was assigned the highest Eigen value of the parasite nodes in the predicted network model, indicating that *G*. *intestinalis* could have been the most easily transmitted zoonotic parasite in the settlement.

### Consuming raw or undercooked infected animal hosts

*Eimeria* spp. and *Perkinsus marinus* are regarded as epizootic diseases that are easily transmitted to hosts that live in close proximity, particularly livestock [34–35]. Neither are zoonotic infections, for instance, birds are the definitive hosts of *E*. *necatrix* whereas bivalves (*Crassostrea* and *Mya*) are the definitive host for *P*. *marinus*, and there is no evidence of *Eimeria* spp. and *P*. *marinus* being pathogenic to humans [36–37]. Bird bones were extracted from the Huecoid (n = 1,185) and Saladoid (n = 1,727) archaeological deposits. Most of these osseous remains were identified as Columbidae in Huecoid (n = 748) and Saladoid (n = 306) deposits. Other bird bones were detected in both deposits, including Rallidae, Pelicanidae, Ardeidae, Anatidae, Phoenicopteridae and Psittacidae among others (S2 Table). Few Psittacidae (Huecoid n = 1 and Saladoid n = 13) remains were found, though they were occasionally consumed, the cultures most likely kept Parrots and Parakeets as pets because of their colorful plumage [4].

*Eimeria* oocysts have been identified in prehistoric ruminant coprolites from Brazil [38]. Clearly *Eimeria* spp. was present in pre-Columbian America, unlike *Perkinsus marinus* which to the best of our knowledge has not been detected in pre-Columbian America samples. *Perkinsus marinus* is highly seasonal, transmission is influence by warmer climates [35]. A variety of bivalve shells were isolated from the deposits, including *Crassostrea*. Huecoid and Saladoid cultures scavenged beach shorelines for bivalves and other forms of small marine life [3]. *Eimeria* spp. and *P*. *marinus* putative reads detected in the metagenomic dataset may be the result of consumption of raw or undercooked infected hosts and thus their presence in the feces may be transient Finding *Eimeria* spp. and *P*. *marinus* in human coprolites provided additional evidence that birds and bivalves were part of their diet.

*Diphyllobothrium* spp. infects fish-eating mammals (definitive host). *Diphyllobothrium* eggs were previously detected in human and canid pre-Columbian coprolites from Peru and Chile [39–40]. Recently *Diphyllobothrium* spp. has been recognized as *Dibothriocephalus* spp., thus *Diphyllobothrium* spp. is a synonymized name. The parasite has two successive intermediate hosts; the first is a copepod and the second a freshwater or marine fish [41–42]. Diphyllobothriidea infection is associated with the ingestion of its raw or undercooked secondary intermediate host [41–42]. In the case of Diphyllobothriidea, cooking the fish kills the plerocercoid larvae in the muscle tissue [41–42], thus the detection of *Diphyllobothrium* spp. eggs could only have occurred by ingesting raw or under cooked infected fish. The Sorcé settlement was 70 meters from the sea, and a variety of marine fish osseous remains were extracted from the Huecoid (n = 1,941) and Saladoid (n = 43,660) archaeological deposits, suggesting both cultures supplemented their diets by fishing [4].

### Transmission by ingestion of intermediate hosts

Canids are the definitive hosts for *Dipylidium caninum*, subsequently canids are reservoirs for the double-pored dog tapeworm. A total of 107 and 168 *Canis familiaris* bones were extracted from the Huecoid and Saladoid deposits (S2 Table), four whole canid remains were found laid out in a burial disposition suggesting endearment towards an animal companion, while scattered canids osseous remains without anatomical context were also dispersed throughout the deposits suggesting canids as occasional food source [4]. Thus far, there is no evidence of *D*. *caninum* human infections associated to the ingestion of infected canids, rather human infections are currently associated with accidental ingestion of its flea vector *Ctenocephalides* spp. [43]. Theoretically, prehistoric cultures may have controlled lice infections by ingesting them while grooming [44–46]. Similar infections have been described in pre-Columbian America in 1,400 year-old coprolites found in Mexico where *Dipylidium* spp. and *Hymenolepis* spp. eggs were detected [10].

*Hymenolepis* spp. sequences were not detected in the metagenomic dataset, however, microscopy suggested the presence of hymenolepidids. Hymenolepididae associated with human infections can also infect rodents, for instance, *H*. *nana* frequently infects humans whereas *H*. *diminuta* infections are uncommon in human hosts [47]. In the case of *H*. *microstoma*, the mouse bile duct tapeworm is questioned as whether to be regarded as a potential zoonotic parasite to humans [48]. Some rodent hymenolepidids are of health interest to humans, since they can cause infections in immunosuppressed individuals [49].

Throughout history, some rodent species have lived as commensals in human settlements [50]. Spanish historians reported that indigenous cultures raised rodents in small corrals [51] to ensure a constant supply of the animal for dietary purposes [4]. Examples include *Isolobodon portoricensis* commonly known as the Puerto Rican Hutia (Huecoid n = 1 and Saladoid n = 323) and the Spiny Rat known as *Heteropsomys insulans* (Huecoid n = 47 and Saladoid n = 19), whose osseous remains were identified in the archaeological deposit (S2 Table). Numerous unidentified rodents bones were also accounted for in Huecoid (n = 151) and Saladoid (n = 50) deposits. It is likely that the rodents that lived in close proximity or within the settlement influenced the transmission of zoonotic infections such as hymenolepidids.

Animal osseous remains found in Sorcé suggest the cultures possible diets, likewise detecting animal and plant aDNA in the gastrointestinal tract (GI) of these individuals is suggestive of their consumption. DNA-based methods have been applied to extant feces to assess the diets of herbivores, carnivorous and omnivorous animals [52–55]. As the ingested tissue passes through the GI tract, DNA from the prey species is substantially degraded [52]. Putative homologous sequences of canids, rodents and fish were observed in the metagenomic datasets (S5 Data). Several putative sequences resulted in a significantly low quality alignment to an animal reference reads which was possibly due to DNA damage inflicted by taphonomical processes [20] and the digestive process of the ingested tissue [52]. A similar study performed metagenomic sequencing of calcified dental plaque of medieval human skeletons, BLASTN alignment to a chloroplast and mitochondrial database revealed putative plant and animal reads [56]. Confirming the putative reads, microscopy examination detected conserved dietary microfossils fragments (such as plant fibers, starch granules, and animal connective tissue) in the dental calculus and zooarchaeological analysis of the medieval site confirmed the presence of animal osseous remains (Suidae, Caprinae, cattle and equids) as the potential protein food source [56]. Detecting putative sequences of animal and plant in the GI tract of ancient cultures via shotgun sequencing is suggestive and must be validated using diverse methods.

### Schistosomatidae

*Schistosoma* spp. has two free-swimming stages that penetrates the intermediate and definitive host skin (Fig 2A and Table 3) [57]. Phylogenetic inference clustered the sequences to Schistosomatidae group. The sequences may correspond to *S*. *mansoni* since the eggs are excreted in the host feces. However, it is possible the sequences may also be associated to another member of the digenean taxon, for example *Trichobilarzia* spp. since it was a close second to *Schistosoma* spp. in a pairwise distance matrix (S11 Table). *Trichobilarzia* spp. definitive hosts are waterfowl, infections have been reported worldwide as migratory waterfowl (such as Anatidae) facilitate the spread of avian schistosomiasis [58–60]. Consequently Anatidae osseous remains were identified in the deposits. *Trichobilarzia* spp. does not mature in humans (accidental host) and instead causes an allergic skin reaction [60–62]. If the cultures ingested the infected tissue of waterfowls, theoretically the parasite would pass through the human GI tract without causing infection. If the sequence is related to *Trichobilarzia* spp., this would consequently alter the network model making canids the principal source of zoonotic infections in the settlement (S4 Table). Trematode eggs were detected by microscopy in a Saladoid coprolite, but could not be identified to the genus level.

*Schistosoma* spp. were not discarded as a potential pathogen. This parasite requires slow-flowing or a still water source for its life cycle to progress and a stream ran near the settlement (Fig 1). Therefore, inhabitants could have potentially acquired the Schistosomatidae and other fresh water related parasites while engaged in activities in the mentioned stream. Low prevalence in wild small mammals (such as rodents) were reported as reservoirs of zoonotic schistosomiasis in West Africa, namely *S*. *mansoni*, *S*. *bovis* and *S*. *haematobium* [63]. If supported, then it could be argued that rodents inhabiting the Sorcé settlement could have been reservoirs for both zoonotic schistosomiasis and Hymenolepid tapeworms. Thus far, ancient *Schistosoma* spp. have mainly been detected in mummies from Egypt and China [14]. To the best of our knowledge *Schistosoma* spp. infections have not been described in pre-Columbian America. Although false taxonomical assignment is a possibility regarding aDNA (see Author’s Statement), we believe that this is not the case seeing as phylogenetic inference strongly suggested the Schistosomatidae group. Thus, if further analyses support these data, this would be the first report of Schistosomatidae in pre-Columbian ethnic groups in America.

## Conclusions

Rodents and canids were probably large contributors of zoonotic infections in pre-Columbian Vieques based on the analyses degree of connectivity and Eigen vector centrality. It is likely that rodents and canids were possible reservoirs of zoonotic infection in the Sorcé settlement. Canids and rodents living in close proximity or inhabiting the settlement could have easily transmitted zoonotic parasites to humans. Dogs could have likely been an occasional food source, whereas the extinct rodent species *Isolobodon portoricensis* (Puerto Rican Hutia) and *Heteropsomys insulans* (Spiny Rat) were an important protein food source for the indigenous cultures on the island. The amount of osseous remains found in these archaeological deposits also suggest that marine fish and birds were also important protein food sources of these cultures.

Polyparasitism was evident in Vieques pre-Columbian cultures [12]. Most of the identified zoonotic parasites could cause gastroenteritis in the infected host. However, some parasites may have been the result of ingestion of the infected animal rather than an actual infection. The coprolites found in Sorcé were intact and well-formed, suggesting asymptomatic infections or perhaps commensal associations between the human hosts and some of the parasites detected in this study. The fact that there were mixed infections present in the coprolites may also indicate that these parasites may become frank pathogens only under certain circumstances and conditions that were not found in the pre-Columbian Antilles. This has been previously hypothesized with other present-day human pathogens detected in ancient cultures [64]. For instance, the Yanomami hunter-gatherer culture in the Amazonian jungle of Venezuela harbor the highest gastrointestinal microbial diversity detected to date in humans [65]. Similar to other semi-isolated indigenous cultures, the intestinal parasite profile of the Yanomami showed evidence of polyparasitisim that can be associated with their life style such as their feeding habits and continued contact with feces contaminated soil [66]. Polyparasitism is frequently associated with underdeveloped areas with poor access to health care and could lead to severe health issues as is the case in rural indigenous communities in South America [67–70]. Although the consistency of fecal samples were not reported in Confalonieri *et al*. [68] and Verhagen *et al*. [70], we suggest that in all future studies of this type the fecal sample characteristics should be reported, as it would be a crucial piece of evidence in the process of determining any asymptomatic parasite infection of these semi-isolated Amerindian cultures. Hypothetically, the detection of well-formed excreta that end up as coprolites could indicate that all infections were either transient (as a result of the ingestion of contaminated food) or that there were indeed commensal polyparasitic "infections" in these pre-Columbian ethnic groups.

## Supporting information

S1 Fig Measuring connectivity with degree of centrality and distribution of the nodes in the network. Degree of centrality measures the amount of nodes connected to neighbor node, a node is important if it has many neighbors. X-axis represents the amount of connectivity (links) and y-axis represent the amount of nodes with said connectivity. Overall, the network has a few nodes that are highly connected representing a real network (power law).
(PDF)

S2 Fig Molecular phylogenetic analysis by maximum likelihood method (BlastX homology search of M01522:132:000000000-A4LNU:1:1110:17795:4053.1). The evolutionary history was inferred by using the Maximum Likelihood method based on the Equal Input model. The bootstrap consensus tree inferred from 1000 replicates.
(PDF)

S3 Fig Molecular phylogenetic analysis by maximum likelihood method (BlastX homology search of M01522:132:000000000-A4LNU:1:1108:20458:16756). The evolutionary history was inferred by using the Maximum Likelihood method based on the Whelan and Goldman model. The bootstrap consensus tree inferred from 1000 replicates.
(PDF)

S4 Fig Molecular phylogenetic analysis by maximum likelihood method (BlastN homology search of M01522:132:000000000-A4LNU:1:1108:20458:16756). The evolutionary history was inferred by using the Maximum Likelihood method based on the Tamura 3-parameter model. The bootstrap consensus tree inferred from 1000 replicates.
(PDF)

S5 Fig Molecular phylogenetic analysis by maximum likelihood method (BlastX homology search of M01522:132:000000000-A4LNU:1:1111:24132:22042.1). The evolutionary history was inferred by using the Maximum Likelihood method based on the Whelan and Goldman model. The bootstrap consensus tree inferred from 1000 replicates.
(PDF)

S6 Fig Molecular phylogenetic analysis by maximum likelihood method (BlastN homology search of M01522:132:000000000-A4LNU:1:1111:24132:22042.1). The evolutionary history was inferred by using the Maximum Likelihood method based on the Tamura 3-parameter model. The bootstrap consensus tree inferred from 1000 replicates.
(PDF)

S7 Fig Molecular phylogenetic analysis by maximum likelihood method (BlastX homology search of M01522:132:000000000-A4LNU:1:1102:17521:21100.1). The evolutionary history was inferred by using the Maximum Likelihood method based on the JTT matrix-based model. The bootstrap consensus tree inferred from 1000 replicates.
(PDF)

S8 Fig Molecular phylogenetic analysis by maximum likelihood method (BlastX homology search of M01522:132:000000000-A4LNU:1:2109:13140:20960.1). The evolutionary history was inferred by using the Maximum Likelihood method based on the Dayhoff matrix based model. The bootstrap consensus tree inferred from 1000 replicates.
(PDF)

S9 Fig Molecular phylogenetic analysis by maximum likelihood method (BlastX homology search of M01522:132:000000000-A4LNU:1:2108:6882:8618). The evolutionary history was inferred by using the Maximum Likelihood method based on the Whelan And Goldman model. The bootstrap consensus tree inferred from 1000 replicates.
(PDF)

S10 Fig Molecular phylogenetic analysis by maximum likelihood method (BlastN homology search of M01522:132:000000000-A4LNU:1:2108:6882:8618). The evolutionary history was inferred by using the Maximum Likelihood method based on the Kimura 2-parameter model. The bootstrap consensus tree inferred from 1000 replicates.
(PDF)

S11 Fig Molecular phylogenetic analysis by maximum likelihood method (BlastX homology search of M01522:132:000000000-A4LNU:1:2110:20683:15891.1). The evolutionary history was inferred by using the Maximum Likelihood method based on the JTT matrix-based model [1]. The bootstrap consensus tree inferred from 1000 replicates.
(PDF)

S12 Fig Molecular phylogenetic analysis by maximum likelihood method (BlastX homology search of M01522:132:000000000-A4LNU:1:2114:18798:18268). The evolutionary history was inferred by using the Maximum Likelihood method based on the JTT matrix-based model. The bootstrap consensus tree inferred from 1000 replicates.
(PDF)

S13 Fig Microscopy images of parasite eggs detected (García Roldán). The two arrows point to two hooks of Hymenolepidid egg.
(PDF)

S1 Table Description of the coprolites used in microscopy examination. (PDF)

S2 Table Faunal osseous remains identified from Huecoid and Saladoid archeological deposits. For modeling purposes, general names for potential parasite host were used, the table uses scientific names and exact count of osseous remains extracted from the Huecoid and Saladoid archeological deposits.
(PDF)

S3 Table Eigen vector centrality of nodes represented in network. A high Eigen vector score means that a node is connected to many nodes which themselves have high scores.
(PDF)

S4 Table Eigen vector centrality if Trichobilharzia node substitutes Schistosoma node in the network. The shift alters the network model making canids the principal contributor of zoonotic infections in the settlement followed by fish, arthopods and lastly rodent nodes.
(PDF)

S5 Table BlastX homologous results of M01522:132:000000000-A4LNU:1:1110:17795:4053.1. (PDF)

S6 Table Estimates of evolutionary divergence between sequences (BlastX M01522:132:000000000-A4LNU:1:1110:17795:4053.1). Analyses were conducted using the JTT matrix-based model.
(PDF)

S7 Table BlastX homologous results of M01522:132:000000000-A4LNU:1:2106:18282:6063.2. BLASTX prediction produced sole homology to *Giardia intestinalis*, thus omitted for phylogenetic analysis.
(PDF)

S8 Table BlastX homologous results of M01522:132:000000000-A4LNU:1:1108:20458:16756. (PDF)

S9 Table Estimates of evolutionary divergence between sequences (BlastX homology search of M01522:132:000000000-A4LNU:1:1108:20458:16756). The number of amino acid substitutions per site from between sequences are shown. Analyses were conducted using the Dayhoff matrix based model.
(PDF)

S10 Table BlastN homologous results of M01522:132:000000000-A4LNU:1:1108:20458:16756. (PDF)

S11 Table Estimates of evolutionary divergence between sequences (BlastN homology search of M01522:132:000000000-A4LNU:1:1108:20458:16756). The number of base substitutions per site from between sequences are shown. Analyses were conducted using the Tamura 3-parameter model.
(PDF)

S12 Table BlastX homologous results of M01522:132:000000000-A4LNU:1:1111:24132:22042.1. (PDF)

S13 Table Estimates of evolutionary divergence between sequences (BlastX homology search of M01522:132:000000000-A4LNU:1:1111:24132:22042.1). The number of amino acid substitutions per site from between sequences are shown. Analyses were conducted using the JTT matrix-based model.
(PDF)

S14 Table BlastN homologous results of M01522:132:000000000-A4LNU:1:1111:24132:22042.1. (PDF)

S15 Table Estimates of evolutionary divergence between sequences (BlastN homology search of M01522:132:000000000-A4LNU:1:1111:24132:22042.1). The number of base substitutions per site from between sequences are shown. Analyses were conducted using the Tamura 3-parameter model.
(PDF)

S16 Table BlastX homologous results of M01522:132:000000000-A4LNU:1:1102:17521:21100.1. (PDF)

S17 Table Estimates of evolutionary divergence between sequences (BlastX homology search of M01522:132:000000000-A4LNU:1:1102:17521:21100.1). The number of amino acid substitutions per site from between sequences are shown. Analyses were conducted using the JTT matrix-based model.
(PDF)

S18 Table BlastX homologous results of M01522:132:000000000-A4LNU:1:2109:13140:20960.1. (PDF)

S19 Table Estimates of evolutionary divergence between sequences (BlastX homology search of M01522:132:000000000-A4LNU:1:2109:13140:20960.1). The number of amino acid substitutions per site from between sequences are shown. Analyses were conducted using the Dayhoff matrix based model.
(PDF)

S20 Table BlastX homologous results of M01522:132:000000000-A4LNU:1:2108:6882:8618. (PDF)

S21 Table Estimates of evolutionary divergence between sequences (BlastX homology search of M01522:132:000000000-A4LNU:1:2108:6882:8618). The number of amino acid substitutions per site from between sequences are shown. Analyses were conducted using the JTT matrix-based model.
(PDF)

S22 Table BlastN homologous results of M01522:132:000000000-A4LNU:1:2108:6882:8618. (PDF)

S23 Table Estimates of evolutionary divergence between sequences (BlastN homology search of M01522:132:000000000-A4LNU:1:2108:6882:8618). The number of base substitutions per site from between sequences are shown. Analyses were conducted using the Kimura 2-parameter model.
(PDF)

S24 Table BlastX homologous results of M01522:132:000000000-A4LNU:1:2110:20683:15891.1. (PDF)

S25 Table Estimates of evolutionary divergence between sequences (BlastX homology search of M01522:132:000000000-A4LNU:1:2110:20683:15891.1). The number of amino acid substitutions per site from between sequences are shown. Analyses were conducted using the JTT matrix-based model.
(PDF)

S26 Table BlastX homologous results of M01522:132:000000000-A4LNU:1:2114:18798:18268. (PDF)

S27 Table Estimates of evolutionary divergence between sequences (BlastX homology search of M01522:132:000000000-A4LNU:1:2114:18798:18268). The number of amino acid substitutions per site from between sequences are shown. Analyses were conducted using the JTT matrix-based model.
(PDF)

S1 Data (XLSX)

S2 Data (XLSX)

S3 Data (XLSX)

S4 Data (XLSX)

S5 Data (R)

S6 Data (ODS)

S7 Data (ODS)
