# Supplementary material for: Pre-Columbian zoonotic enteric parasites: An insight into Puerto Rican indigenous culture diets and life styles
Source: PLoS One. 2020 Jan 30;15(1):e0227810. doi: 10.1371/journal.pone.0227810 (PMC6992007; doi:10.1371/journal.pone.0227810)
Supplement: S1 Fig — Degree of centrality measures the amount of nodes connected to neighbor node, a node is important if it has many neighbors. X-axis represents the amount of connectivity (links) and y-axis represent the amount of nodes with said connectivity. Overall, the network has a few nodes that are highly connected representing a real network (power law). (PDF) [file pone.0227810.s001.pdf]

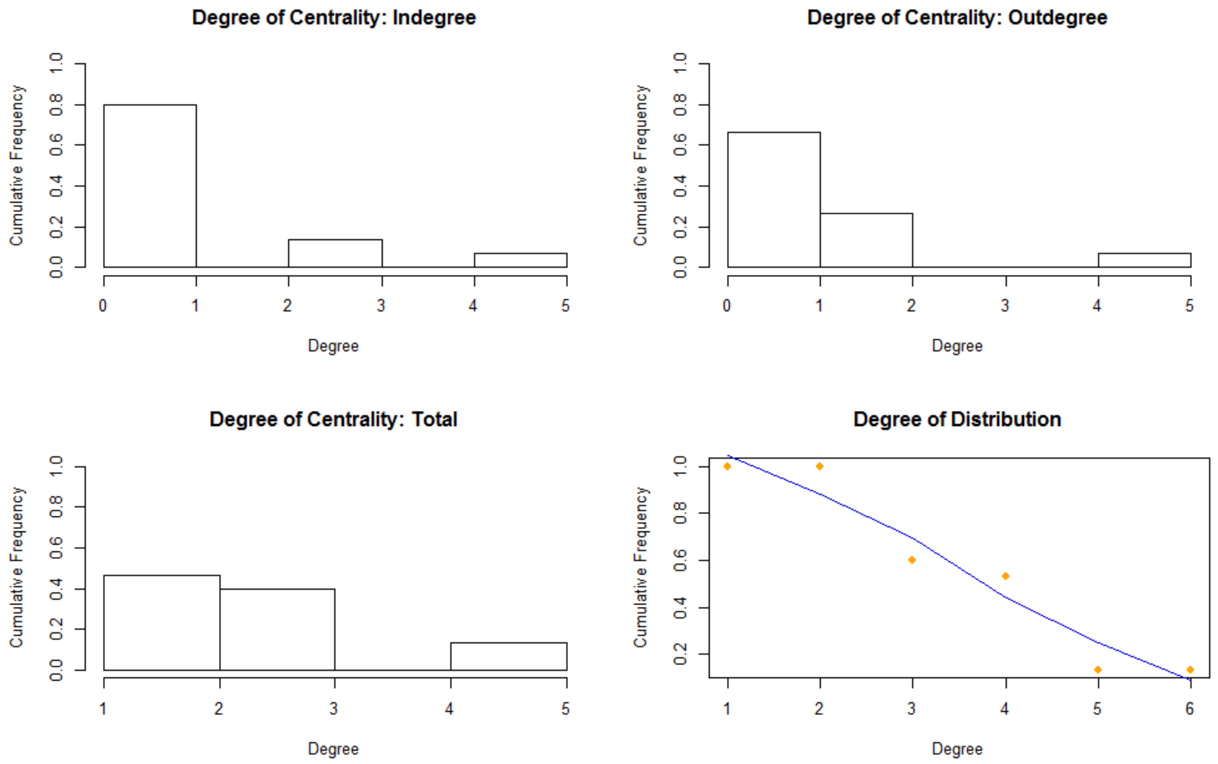

S1 Fig. Measuring connectivity with degree of centrality and distribution of the nodes in the network. Degree of centrality measures the amount of nodes connected to neighbor node, a node is important if it has many neighbors. X-axis represents the amount of connectivity (links) and y-axis represent the amount of nodes with said connectivity. Overall, the network has a few nodes that are highly connected representing a real network (power law).
