# Supplementary material for: Pre-Columbian zoonotic enteric parasites: An insight into Puerto Rican indigenous culture diets and life styles
Source: PLoS One. 2020 Jan 30;15(1):e0227810. doi: 10.1371/journal.pone.0227810 (PMC6992007; doi:10.1371/journal.pone.0227810)
Supplement: S9 Fig — The evolutionary history was inferred by using the Maximum Likelihood method based on the Whelan And Goldman model. The bootstrap consensus tree inferred from 1000 replicates. (PDF) [file pone.0227810.s009.pdf]

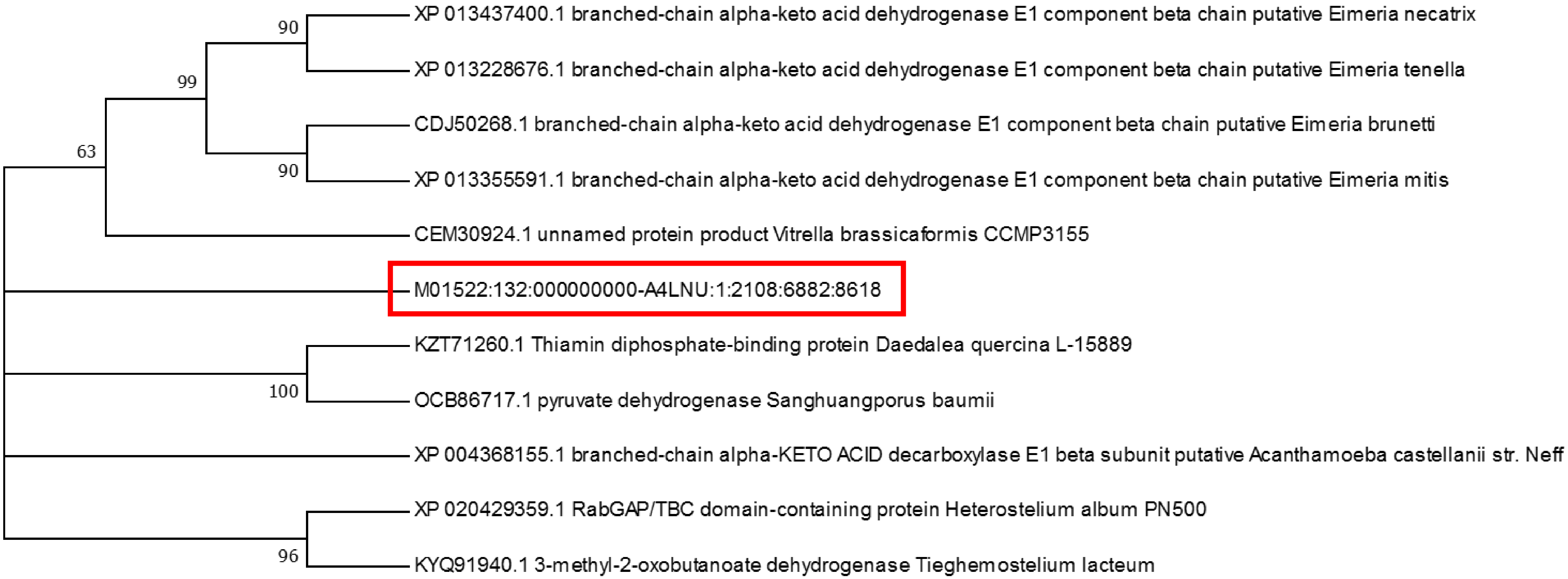

S9 Fig. Molecular Phylogenetic analysis by Maximum Likelihood method (BlastX homology search of M01522:132:000000000-A4LNU:1:2108:6882:8618). The evolutionary history was inferred by using the Maximum Likelihood method based on the Whelan And Goldman model. The bootstrap consensus tree inferred from 1000 replicates.
