# Supplementary material for: Pre-Columbian zoonotic enteric parasites: An insight into Puerto Rican indigenous culture diets and life styles
Source: PLoS One. 2020 Jan 30;15(1):e0227810. doi: 10.1371/journal.pone.0227810 (PMC6992007; doi:10.1371/journal.pone.0227810)
Supplement: S13 Fig — The two arrows point to two hooks of Hymenolepidid egg. (PDF) [file pone.0227810.s013.pdf]

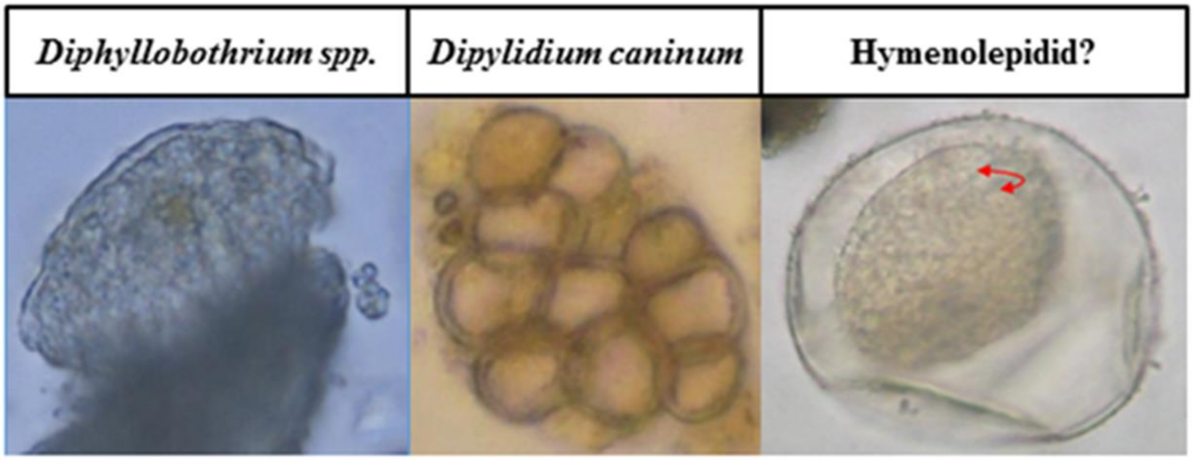

S13 Fig. Microscopy images of parasite eggs detected (García Roldán). The two arrows point to two hooks of Hymenolepidid egg**.**
