# Supplementary material for: Pre-Columbian zoonotic enteric parasites: An insight into Puerto Rican indigenous culture diets and life styles
Source: PLoS One. 2020 Jan 30;15(1):e0227810. doi: 10.1371/journal.pone.0227810 (PMC6992007; doi:10.1371/journal.pone.0227810)
Supplement: S1 Table — (PDF) [file pone.0227810.s014.pdf]

S1 Table. Description of the coprolites used in microscopy examination.

Registration	Culture	Deposit	Quadrant	Depth
**5.2003.0001**	Huecoid	Z	Z - 20	2.00 mt.
**5.2003.0002**	Huecoid	Z	Z - 37	0.20-0.40 cm.
**5.2003.0003**	Huecoid	Z	Z - C	2.60 mt.
**5.2003.0004**	Huecoid	Z	Z - C	2.40 mt.
**5.2003.0005**	Huecoid	Z	Z - C	1.80 mt.
**5.2003.0006**	Huecoid	Z	Z - L	0.70 cm.
**5.2003.0007**	Huecoid	Z	Z - M	1.20 mt.
**5.2003.0008**	Huecoid	Z	Z - V	2.20 mt.
**5.2003.0009**	Huecoid	Z	Z - W	1.60 mt.
**5.2003.0010**	Huecoid	Z	Z - W	1.80 mt.
**5.2003.0011**	Huecoid	Z	Z - W	2.00 mt.
**5.2003.0012**	Huecoid	Z	Z - X	0.60 cm.
**5.2003.0013**	Huecoid	Z	Z - Z	1.40 mt
**5.2003.0014**	Saladoid	YTA-2	J - 22	0.80 cm.
**5.2003.0015**	Saladoid	YTA-2	H - 21	1.20 mt.
**5.2003.0016**	Saladoid	YTA-2	M - 15	0.40 cm.
**5.2003.0017**	Saladoid	YTA-2	I - 15	1.00 mt.
**5.2003.0018**	Saladoid	YTA-2	I - 24	1.00 - 1.20 mt.
**5.2003.0019**	Saladoid	YTA-1	I - 5	0.60 cm.
**5.2003.0020**	Saladoid	ZT	S - 1	0.90 cm.

*The rows colored yellow refer to the coprolites that were in both the microscopic and in the molecular analysis.
