# Supplementary material for: Pre-Columbian zoonotic enteric parasites: An insight into Puerto Rican indigenous culture diets and life styles
Source: PLoS One. 2020 Jan 30;15(1):e0227810. doi: 10.1371/journal.pone.0227810 (PMC6992007; doi:10.1371/journal.pone.0227810)
Supplement: S2 Table — For modeling purposes, general names for potential parasite host were used, the table uses scientific names and exact count of osseous remains extracted from the Huecoid and Saladoid archeological deposits. (PDF) [file pone.0227810.s015.pdf]

S2 Table. Faunal osseous remains identified from Huecoid and Saladoid archeological deposits. For modeling purposes, general names for potential parasite host were used, the table uses scientific names and exact count of osseous remains extracted from the Huecoid and Saladoid archeological deposits.

Group	Clasification	Potential Host	Description	Huecoid	Saladoid
Birds	Family	Sulidae	Boobies	3	9
		Pelecanidae	Pelicans	1	3
		Fregatidae	Frigatebirds	1	6
		Ardeidae	Herons, Bitterns, and Egrets	26	31
		Anatidae	Swans, Geese, and Duck	2	32
		Arserinae	Geese	2	-
		Phoenicopteridae	Flamingo	3	54
		Accipitriformes	Hawk	-	9
		Flaconidae	Falcons	2	3
		Rallidae	Rails, Gallinules, and Coots	6	30
		Laridae	Gulls, Terns and Skimmers	2	2
		Columbidae	Pigeons and Doves	748	306
		Psittacidae	Parrots and Parakeets	1	13
		Corvidae	Crows, Magpies, and Jays	4	-
		Muscicapidae	Thrush	20	-
	Unidentified Birds			364	1229
Reptiles	Family	Cheloniidae	Sea Turtle	27	2231
	Genus	*Anolis spp.*	Lizard	-	1
	Species	*Cyclura spp.*	Iguana	35	1
		Ameiva exsul	Puerto Rican Ground Lizard	-	30
		Alsophis portoricensis	Snake	-	5
		Epicrates inornatus	Boa	-	136
		Trachemys stejnegeri	Land Turtle	4	83
	Unidentified Reptiles			2	32
Fish	Family	Carcharhinidae	Sharks	28	8
		Dasyatidae	Stingray	7	1
		Megalopidae	Tarpon	6	18
		Albulidae	Bonefish	1	21
		Belonidae	Needlefish	11	130
		Centropomidae	Snooks	8	595
		Serranidae	Groupers	274	2234
		Lutjanidae	Snappers	71	817
		Carangidae	Jacks	26	347
		Gerreidae	Mojarras	-	44
		Haemulidae	Grunts	37	1127
		Sparidae	Porgies and Parrotfish	17	950
		Sciaenidae	Drums	2	1
		Sphyraenidae	Barracuda	20	8
		Labridae	Hogfish and Wrasse	53	257
		Scombridae	Tunas and Mackerels	1	2272
		Diodontidae	Porcupinefish	4	1518
		Balistidae	Triggerfish	17	422
	Unidentified Fish			1358	32890

**Mammals**	**Species**	*Heteropsomys insulans*	Spiny Rat	47	19
		Isolobodon portoricensis	Puerto Rican Hutia	1	323
	Unidentified Rodent			151	50
	**Family**	Delphinidae	Dolphins and Porpoises	1	-
	**Species**	*Nesophontes edithae*	Puerto Rican Shrew	6	28
		Canis familiaris	Canid	107	168
		Trichechus manatus	Manatee	7	1
	Unidentified Mammals			42	35
