# Supplementary material for: Pre-Columbian zoonotic enteric parasites: An insight into Puerto Rican indigenous culture diets and life styles
Source: PLoS One. 2020 Jan 30;15(1):e0227810. doi: 10.1371/journal.pone.0227810 (PMC6992007; doi:10.1371/journal.pone.0227810)
Supplement: S3 Table — A high Eigen vector score means that a node is connected to many nodes which themselves have high scores. (PDF) [file pone.0227810.s016.pdf]

S3 Table. Eigen vector centrality of nodes represented in network. A high Eigen vector score means that a node is connected to many nodes which themselves have high scores.

Eigen Vector Centrality of Nodes in Network		
	**Nodes**	**Eigen Value**
**Parasite**	*G. intestinalis*	0.89
	Diphyllobothrium	0.06
	D. caninum	0.18
	Hymenolepis	0.18
	Schistosoma	0.18
**Host**	Birds	0.28
	Canids	0.64
	Copepod	0.20
	Fish	0.58
	Humans	1.00
	Pulicidae	0.57
	Arthropod	0.57
	Gastropods	0.57
	Reptiles	0.28
	Rodents	0.64
