# Supplementary material for: Pre-Columbian zoonotic enteric parasites: An insight into Puerto Rican indigenous culture diets and life styles
Source: PLoS One. 2020 Jan 30;15(1):e0227810. doi: 10.1371/journal.pone.0227810 (PMC6992007; doi:10.1371/journal.pone.0227810)
Supplement: S4 Table — The shift alters the network model making canids the principal contributor of zoonotic infections in the settlement followed by fish, arthopods and lastly rodent nodes. (PDF) [file pone.0227810.s017.pdf]

S4 Table. Eigen vector centrality if *Trichobilharzia* node substitutes *Schistosoma* node in the network. The shift alters the network model making canids the principal contributor of zoonotic infections in the settlement followed by fish, arthopods and lastly rodent nodes.

Eigen Vector Centrality of Nodes in Network		
	**Nodes**	**Eigen Value**
**Parasite**	*G. intestinalis*	1.00
	Diphyllobothrium	0.08
	D. caninum	0.22
	Hymenolepis	0.19
	Trichobilharzia	0.05
**Host**	Birds	0.38
	Canids	0.78
	Copepod	0.25
	Fish	0.67
	Humans	0.97
	Pulicidae	0.66
	Arthropod	0.56
	Gastropods	0.14
	Reptiles	0.33
	Rodents	0.52
