# Supplementary material for: Pre-Columbian zoonotic enteric parasites: An insight into Puerto Rican indigenous culture diets and life styles
Source: PLoS One. 2020 Jan 30;15(1):e0227810. doi: 10.1371/journal.pone.0227810 (PMC6992007; doi:10.1371/journal.pone.0227810)
Supplement: S5 Table — (PDF) [file pone.0227810.s018.pdf]

S5 Table. BlastX homologous results of M01522:132:000000000-A4LNU:1:1110:17795:4053.1

Sequence ID	Specie ID	Max Score	Total Score	Query Cover	E-Value	Identification	Accession
M01522:132:000000000-A4LNU:1:1110:17795:4053.1	heat shock protein 70 [Cryptosporidium sp.]	119	119	100%	2E-33	79%	AAL84005.1
	heat shock 70 kDa protein [Thraustotheca clavata]	117	117	100%	3E-29	79%	OQR88359.1
	hypothetical protein AZE42_13959 [Rhizopogon vesiculosus]	110	110	100%	4E-29	75%	OJA14560.1
	hsp70-like protein [Phytophthora parasitica P1976]	112	112	100%	3E-28	75%	ETO79273.1
	heat shock protein 70kDa [Salpingoeca rosetta]	114	114	100%	5E-28	78%	XP_004987893.1
	hsp70-like protein [Phytophthora parasitica P10297]	112	112	100%	5E-28	75%	ETP48231.1
	heat shock 70 kDa protein [Phytophthora infestans T30-4]	114	114	100%	5E-28	75%	XP_002902006.1
	heat shock protein [Salpingoeca rosetta]	114	114	100%	7E-28	78%	XP_004997347.1
	hsp70-like protein [Phytophthora parasitica]	112	112	100%	9E-28	75%	ETL96981.1
	heat shock 70 kDa protein [Phytophthora infestans T30-4]	113	113	100%	1E-27	75%	XP_002902008.1
