# Supplementary material for: Pre-Columbian zoonotic enteric parasites: An insight into Puerto Rican indigenous culture diets and life styles
Source: PLoS One. 2020 Jan 30;15(1):e0227810. doi: 10.1371/journal.pone.0227810 (PMC6992007; doi:10.1371/journal.pone.0227810)
Supplement: S6 Table — Analyses were conducted using the JTT matrix-based model. (PDF) [file pone.0227810.s019.pdf]

S6 Table. Estimates of Evolutionary Divergence between Sequences (BlastX M01522:132:000000000-A4LNU:1:1110:17795:4053.1).
Analyses were conducted using the JTT matrix-based model.

		1	2	3	4	5	6	7	8	9	10	11
**M01522:132:000000000-A4LNU:1:1110:17795:4053.1**	**1**											
**AAL84005.1_heat_shock_protein_70_partial_Cryptosporidium_sp.**	**2**	0.14										
**OQR88359.1_heat_shock_70_kDa_protein_partial_Thraustotheca_clavata**	**3**	0.23	0.28									
**OJA14560.1_hypothetical_protein_AZE42_13959_partial_Rhizopogon_vesiculosus**	**4**	0.30	0.34	0.32								
**ETO79273.1_hsp70-like_protein_partial_Phytophthora_parasitica_P1976**	**5**	0.29	0.33	0.17	0.32							
**XP_004987893.1_heat_shock_protein_70kDa_Salpingoeca_rosetta**	**6**	0.25	0.31	0.25	0.36	0.27						
**ETP48231.1_hsp70-like_protein_partial_Phytophthora_parasitica_P10297**	**7**	0.29	0.33	0.17	0.32	0.00	0.27					
**XP_002902006.1_heat_shock_70_kDa_protein_Phytophthora_infestans_T30-4**	**8**	0.29	0.35	0.16	0.33	0.06	0.29	0.06				
**XP_004997347.1_heat_shock_protein_Salpingoeca_rosetta**	**9**	0.25	0.31	0.25	0.36	0.27	0.00	0.27	0.29			
**ETL96981.1_hsp70-like_protein_partial_Phytophthora_parasitica**	**10**	0.29	0.33	0.17	0.32	0.00	0.27	0.00	0.06	0.27		
**XP_002902008.1_heat_shock_70_kDa_protein_Phytophthora_infestans_T30-4**	**11**	0.29	0.35	0.16	0.33	0.04	0.29	0.04	0.01	0.29	0.04	
