# Supplementary material for: Pre-Columbian zoonotic enteric parasites: An insight into Puerto Rican indigenous culture diets and life styles
Source: PLoS One. 2020 Jan 30;15(1):e0227810. doi: 10.1371/journal.pone.0227810 (PMC6992007; doi:10.1371/journal.pone.0227810)
Supplement: S7 Table — BLASTX prediction produced sole homology to Giardia intestinalis, thus omitted for phylogenetic analysis. (PDF) [file pone.0227810.s020.pdf]

S7 Table. BlastX homologous results of M01522:132:000000000-A4LNU:1:2106:18282:6063.2. BLASTX prediction produced sole homology to *Giardia intestinalis*, thus omitted for phylogenetic analysis.

	Specie ID	Max Score	Total Score	Query Cover	E-Value	Identification	Accession
M01522:132:000000000-A4LNU:1:2106:18282:6063.2	glutamate dehydrogenase [Giardia intestinalis]	124	124	97%	1e-35	85%	BAH19351.1
	glutamate dehydrogenase [Giardia intestinalis]	124	124	97%	1e-35	85%	AEW29656.1
	glutamate dehydrogenase [Giardia intestinalis]	124	124	97%	1e-35	85%	AEW29649.1
	glutamate dehydrogenase [Giardia intestinalis]	124	124	97%	2e-35	85%	ALQ81510.1
	glutamate dehydrogenase [Giardia intestinalis]	124	124	97%	2e-35	85%	AOZ21456.1
	glutamate dehydrogenase [Giardia intestinalis]	124	124	97%	3e-35	85%	AVC68692.1
	glutamate dehydrogenase [Giardia intestinalis]	124	124	97%	3e-35	85%	AVC68685.1
	glutamate dehydrogenase [Giardia intestinalis]	123	123	97%	3e-35	85%	AFK80368.1
	glutamate dehydrogenase [Giardia intestinalis]	124	124	97%	3e-35	85%	AAV86073.1
	glutamate dehydrogenase [Giardia intestinalis]	124	124	97%	3e-35	85%	AHZ90122.1
	glutamate dehydrogenase [Giardia intestinalis]	124	124	97%	3.00E-35	85%	AVC68681.1
	glutamate dehydrogenase [Giardia intestinalis]	124	124	97%	3.00E-35	85%	AMN92209.1
	glutamate dehydrogenase [Giardia intestinalis]	123	123	97%	4.00E-35	85%	AFK80367.1
	glutamate dehydrogenase [Giardia intestinalis]	124	124	97%	4.00E-35	85%	AHZ90126.1
	glutamate dehydrogenase [Giardia intestinalis]	123	123	97%	4.00E-35	85%	AFK80365.1
	glutamate dehydrogenase [Giardia intestinalis]	124	124	97%	4.00E-35	85%	AVC68688.1
	glutamate dehydrogenase [Giardia intestinalis]	124	124	97%	4.00E-35	85%	AHZ90127.1
	glutamate dehydrogenase [Giardia intestinalis]	124	124	97%	4.00E-35	85%	AVC68684.1
	glutamate dehydrogenase [Giardia intestinalis]	124	124	97%	4.00E-35	85%	AVC68683.1
	glutamate dehydrogenase [Giardia intestinalis]	124	124	97%	4.00E-35	85%	AVC68686.1
	glutamate dehydrogenase [Giardia intestinalis]	124	124	97%	5.00E-35	85%	AVC68687.1
	glutamate dehydrogenase [Giardia intestinalis]	124	124	97%	5.00E-35	85%	AHZ90125.1
	glutamate dehydrogenase [Giardia intestinalis]	124	124	97%	5.00E-35	85%	ABG78271.1
	glutamate dehydrogenase [Giardia intestinalis]	123	123	97%	5.00E-35	83%	AEW29657.1
	glutamate dehydrogenase [Giardia intestinalis]	124	124	97%	5.00E-35	85%	AHZ90123.1
	glutamate dehydrogenase [Giardia intestinalis]	124	124	97%	5.00E-35	85%	AHZ90124.1
	glutamate dehydrogenase [Giardia intestinalis]	124	124	97%	5.00E-35	85%	AVC68680.1
	glutamate dehydrogenase [Giardia intestinalis]	124	124	97%	5.00E-35	85%	AVC68682.1
	glutamate dehydrogenase [Giardia intestinalis]	123	123	97%	6.00E-35	85%	AVC68689.1
	NADP-dependent glutamate dehydrogenase [Giardia intestinalis]	124	124	97%	6.00E-35	85%	ANC51918.1
	NADP-dependent glutamate dehydrogenase [Giardia intestinalis]	124	124	97%	6.00E-35	85%	AFU66027.1
	glutamate dehydrogenase [Giardia intestinalis]	124	124	97%	6.00E-35	85%	AMN92200.1
	glutamate dehydrogenase [Giardia intestinalis]	122	122	97%	6.00E-35	83%	AEW29661.1
	NADP-dependent glutamate dehydrogenase [Giardia intestinalis]	124	124	97%	6.00E-35	85%	AFU66029.1
	glutamate dehydrogenase [Giardia intestinalis]	123	123	97%	6.00E-35	83%	BAK52282.1
	glutamate dehydrogenase [Giardia intestinalis]	124	124	97%	6.00E-35	85%	ACF05220.1
	NADP-dependent glutamate dehydrogenase [Giardia intestinalis]	124	124	97%	6.00E-35	85%	ASH97612.1
	NADP-dependent glutamate dehydrogenase [Giardia intestinalis]	124	124	97%	6.00E-35	85%	AFU66022.1
	glutamate dehydrogenase [Giardia sp.]	124	124	97%	6.00E-35	85%	ARL00180.1
	NADP-dependent glutamate dehydrogenase [Giardia intestinalis]	124	124	97%	6.00E-35	85%	AQT40982.1
	glutamate dehydrogenase [Giardia intestinalis]	122	122	97%	6.00E-35	83%	ANF07107.1
	NADP-dependent glutamate dehydrogenase [Giardia intestinalis]	124	124	97%	7.00E-35	85%	AFU66028.1
	NADP-dependent glutamate dehydrogenase [Giardia intestinalis]	124	124	97%	7.00E-35	85%	ASH97613.1
	NADP-dependent glutamate dehydrogenase [Giardia intestinalis]	124	124	97%	7.00E-35	85%	AFU66021.1
	NADP-dependent glutamate dehydrogenase [Giardia intestinalis]	124	124	97%	7.00E-35	85%	ABD66001.1
	glutamate dehydrogenase [Giardia intestinalis]	123	123	97%	7.00E-35	83%	AQY61800.1
	NADP-dependent glutamate dehydrogenase [Giardia intestinalis]	124	124	97%	7.00E-35	85%	AUS84254.1
	glutamate dehydrogenase [Giardia intestinalis]	122	122	97%	7.00E-35	85%	ALX30933.1
