# Supplementary material for: Pre-Columbian zoonotic enteric parasites: An insight into Puerto Rican indigenous culture diets and life styles
Source: PLoS One. 2020 Jan 30;15(1):e0227810. doi: 10.1371/journal.pone.0227810 (PMC6992007; doi:10.1371/journal.pone.0227810)
Supplement: S8 Table — (PDF) [file pone.0227810.s021.pdf]

S8 Table. BlastX homologous results of M01522:132:000000000-A4LNU:1:1108:20458:16756

	Specie ID	Max Score	Total Score	Query Cover	E-Value	Identification	Accession
M01522:132:000000000-A4LNU:1:1108:20458:16756	dolichyl glycosyltransferase [Schistosoma mansoni]	117	117	0.71	4e-28	0.57	XP_018649914.1
	dolichyl glycosyltransferase [Schistosoma haematobium]	116	116	0.7	4e-28	0.57	XP_012800852.1
	hypotherical protein [Schistosoma japonicum]	114	114	0.71	5e-27	0.56	CAX74464.1
	ALG6, ALG8 glycosyltransferase family protein [Trichuris suis]	113	113	0.7	5e-27	0.56	KHJ44037.1
	hypothetical protein M514_00337 [Trichuris suis]	113	113	0.7	8e-27	0.56	KFD68608.1
	hypothetical protein M513_00337 [Trichuris suis]	112	112	0.7	9e-27	0.56	KFD58644.1
	ALG6, ALG8 glycosyltransferase family protein [Fasciola hepatica]	112	112	0.7	2e-26	0.55	PIS90652.1
	PREDICTED: dolichyl glycosyltransferase [Priapulus caudatus]	112	112	0.71	4e-26	0.55	XP_014673327.1
	hypothetical protein WR25_21702 [Diploscapter pachys]	110	110	0.7	5e-26	0.57	PAV83949.1
	hypotherical protein [Schistosoma japonicum]	110	110	0.71	7e-26	0.55	CAX74463.1
