# Supplementary material for: Pre-Columbian zoonotic enteric parasites: An insight into Puerto Rican indigenous culture diets and life styles
Source: PLoS One. 2020 Jan 30;15(1):e0227810. doi: 10.1371/journal.pone.0227810 (PMC6992007; doi:10.1371/journal.pone.0227810)
Supplement: S9 Table — The number of amino acid substitutions per site from between sequences are shown. Analyses were conducted using the Dayhoff matrix based model. (PDF) [file pone.0227810.s022.pdf]

S9 Table. Estimates of Evolutionary Divergence between Sequences (BlastX homology search of M01522:132:000000000-A4LNU:1:1108:20458:16756). The number of amino acid substitutions per site from between sequences are shown. Analyses were conducted using the Dayhoff matrix based model.

		1	2	3	4	5	6	7	8	9	10
XP_018649914.1_dolichyl_glycosyltransferase_Schistosoma_mansoni	1										
XP_012800852.1_dolichyl_glycosyltransferase_Schistosoma_haematobium	2	0.08									
M01522:132:000000000-A4LNU:1:1108:20458:16756	3	0.93	0.87								
CAX74464.1_hypotherical_protein_Schistosoma_japonicum	4	0.17	0.15	0.95							
KHJ44037.1_ALG6_ALG8_glycosyltransferase_family_protein_Trichuris_suis	5	0.59	0.56	0.94	0.59						
KFD68608.1_hypothetical_protein_M514_00337_Trichuris_suis	6	0.59	0.56	0.94	0.59	0.00					
KFD58644.1_hypothetical_protein_M513_00337_Trichuris_suis	7	0.59	0.56	0.94	0.59	0.00	0.00				
PIS90652.1_ALG6_ALG8_glycosyltransferase_family_protein_Fasciola_hepatica	8	0.44	0.43	0.93	0.44	0.66	0.66	0.66			
PAV83949.1_hypothetical_protein_WR25_21702_Diploscapter_pachys	9	0.51	0.55	0.94	0.54	0.45	0.45	0.45	0.54		
CAX74463.1_hypotherical_protein_Schistosoma_japonicum	10	0.19	0.17	0.98	0.01	0.62	0.62	0.62	0.47	0.57	
