# Supplementary material for: Pre-Columbian zoonotic enteric parasites: An insight into Puerto Rican indigenous culture diets and life styles
Source: PLoS One. 2020 Jan 30;15(1):e0227810. doi: 10.1371/journal.pone.0227810 (PMC6992007; doi:10.1371/journal.pone.0227810)
Supplement: S10 Table — (PDF) [file pone.0227810.s023.pdf]

S10 Table. BlastN homologous results of M01522:132:000000000-A4LNU:1:1108:20458:16756

	Specie ID	Max Score	Total Score	Query Cover	E-Value	Identification	Accession
M01522:132:000000000-A4LNU:1:1108:20458:16756	Schistosoma mansoni dolichyl glycosyltransferase partial mRNA	125	125	45%	9.00E-25	76%	XM_018795619.1
	Schistosoma rodhaini genome assembly S_rodhaini_Burundi	125	125	45%	9.00E-25	76%	LL957621.1
	Schistosoma mansoni strain Puerto Rico chromosome 2, complete genome	125	125	45%	9.00E-25	76%	HE601625.1
	Schistosoma mattheei genome assembly S_mattheei_Denwood	120	120	46%	4.00E-23	76%	LM158041.1
	Schistosoma curassoni genome assembly S_curassoni_Dakar	116	116	46%	4.00E-22	76%	LM076444.1
	Schistosoma haematobium Dolichyl pyrophosphate	113	113	46%	5.00E-21	75%	XM_012945398.1
	Schistosoma japonicum isolate Anhui full length mRNA clone SJFCE2873.	111	111	45%	2.00E-20	75%	FN318736.1
	Schistosoma japonicum isolate Anhui full length mRNA clone SJFCE2873.	107	107	45%	2.00E-19	74%	FN318735.1
	Schistosoma japonicum SJCHGC03673 protein mRNA	105	105	45%	8.00E-19	74%	AY810735.1
	Trichobilharzia regenti genome assembly T_regenti_v1_0_4	104	104	43%	3.00E-18	75%	LL014407.1
