# Supplementary material for: Pre-Columbian zoonotic enteric parasites: An insight into Puerto Rican indigenous culture diets and life styles
Source: PLoS One. 2020 Jan 30;15(1):e0227810. doi: 10.1371/journal.pone.0227810 (PMC6992007; doi:10.1371/journal.pone.0227810)
Supplement: S11 Table — The number of base substitutions per site from between sequences are shown. Analyses were conducted using the Tamura 3-parameter model. (PDF) [file pone.0227810.s024.pdf]

S11 Table. Estimates of Evolutionary Divergence between Sequences (BlastN homology search of M01522:132:000000000-A4LNU:1:1108:20458:16756). The number of base substitutions per site from between sequences are shown. Analyses were conducted using the Tamura 3-parameter model.

		1	2	3	4	5	6	7	8	9	10	11
M01522:132:000000000-A4LNU:1:1108:20458:16756	1											
XM_018795619.1_Schistosoma_mansoni_dolichyl_glycosyltransferase_partial_mRNA	2	0.86										
LL957621.1_Schistosoma_rodhaini_genome_assembly	3	0.30	0.87									
HE601625.1_Schistosoma_mansoni_strain_Puerto_Rico_chromosome_2	4	0.29	0.90	0.03								
LM158041.1_Schistosoma_mattheei_genome_assembly	5	0.87	0.07	0.89	0.91							
LM076444.1_Schistosoma_curassoni_genome_assembly	6	0.85	0.06	0.86	0.89	0.01						
XM_012945398.1_Schistosoma_haematobium_Dolichyl_pyrophosphate	7	0.87	0.08	0.84	0.86	0.02	0.01					
FN318736.1_Schistosoma_japonicum_isolate_Anhui_full_length_mRNA_clone_SJFCE2873.003	8	0.79	0.16	0.80	0.82	0.17	0.16	0.17				
FN318735.1_Schistosoma_japonicum_isolate_Anhui_full_length_mRNA_clone_SJFCE2873.002	9	0.80	0.17	0.80	0.83	0.18	0.17	0.18	0.01			
AY810735.1_Schistosoma_japonicum_SJCHGC03673	10	0.80	0.17	0.80	0.83	0.18	0.17	0.18	0.01	0		
LL014407.1_Trichobilharzia_regenti_genome_assembly	11	0.31	0.85	0.21	0.19	0.89	0.86	0.84	0.84	0.84	0.84	
