# Supplementary material for: Pre-Columbian zoonotic enteric parasites: An insight into Puerto Rican indigenous culture diets and life styles
Source: PLoS One. 2020 Jan 30;15(1):e0227810. doi: 10.1371/journal.pone.0227810 (PMC6992007; doi:10.1371/journal.pone.0227810)
Supplement: S12 Table — (PDF) [file pone.0227810.s025.pdf]

S12 Table. BlastX homologous results of M01522:132:000000000-A4LNU:1:1111:24132:22042.1

	Specie ID	Max Score	Total Score	Query Cover	E-Value	Identification	Accession
M01522:132:000000000-A4LNU:1:1111:24132:22042.1	Dolichyl pyrophosphate [Schistosoma haematobium]	106	106	0.98	3E-25	0.57	XP_012800852.1
	ALG6, ALG8 glycosyltransferase family protein [Fasciola hepatica]	105	105	0.98	8e-25	0.56	PIS90652.1
	dolichyl glycosyltransferase [Schistosoma mansoni]	105	105	0.98	2e-24	0.57	XP_018649914.1
	hypotherical protein [Schistosoma japonicum]	101	101	0.98	4e-23	0.55	CAX74464.1
	PREDICTED: probable dolichyl pyrophosphate [Rhagoletis zephyria]	100	100	0.98	5e-23	0.55	XP_017479008.1
	probable dolichyl pyrophosphate [Pseudomyrmex gracilis]	99.8	99.8	0.98	1e-22	0.52	XP_020297982.1
	PREDICTED: dolichyl pyrophosphate [Ciona intestinalis]	99.8	99.8	0.98	1e-22	0.55	XP_002129715.1
	ALG6, ALG8 glycosyltransferase family protein [Trichuris suis]	99.4	99.4	0.98	1e-22	0.54	KHJ44037.1
	PREDICTED: probable dolichyl pyrophosphate [Ooceraea biroi]	99.8	99.8	0.98	2e-22	0.54	XP_019890096.1
	dolichyl glycosyltransferase, putative [Pediculus humanus corporis]	99	99.0	0.98	2e-22	0.55	XP_002429694.1
