# Supplementary material for: Pre-Columbian zoonotic enteric parasites: An insight into Puerto Rican indigenous culture diets and life styles
Source: PLoS One. 2020 Jan 30;15(1):e0227810. doi: 10.1371/journal.pone.0227810 (PMC6992007; doi:10.1371/journal.pone.0227810)
Supplement: S13 Table — The number of amino acid substitutions per site from between sequences are shown. Analyses were conducted using the JTT matrix-based model. (PDF) [file pone.0227810.s026.pdf]

S13 Table. Estimates of Evolutionary Divergence between Sequences (BlastX homology search of M01522:132:000000000-A4LNU:1:1111:24132:22042.1). The number of amino acid substitutions per site from between sequences are shown. Analyses were conducted using the JTT matrix-based model.

		1	2	3	4	5	6	7	8	9	10	11
M01522:132:000000000-A4LNU:1:1111:24132:22042.1	1											
XP_012800852.1_Dolichyl_pyrophosphate_Schistosoma_haematobium	2	1.57										
PIS90652.1_ALG6_ALG8_glycosyltransferase_family_protein_Fasciola_hepatica	3	1.66	0.58									
XP_018649914.1_dolichyl_glycosyltransferase_Schistosoma_mansoni	4	1.60	0.09	0.57								
CAX74464.1_hypotherical_protein_Schistosoma_japonicum	5	1.92	0.19	0.54	0.19							
XP_017479008.1_PREDICTED:_probable_dolichyl_Rhagoletis_zephyria	6	1.92	1.76	1.48	1.88	1.63						
XP_020297982.1_probable_dolichyl_pyrophosphate_Pseudomyrmex_gracilis	7	2.20	1.06	1.05	1.08	1.10	0.98					
XP_002129715.1_PREDICTED:_dolichyl_pyrophosphate_Ciona_intestinalis	8	1.90	0.91	1.27	0.71	0.99	1.18	0.95				
KHJ44037.1_ALG6_ALG8_glycosyltransferase_family_protein_Trichuris_suis	9	1.89	0.9	1.07	0.97	1.08	1.43	1.17	1.21			
XP_019890096.1_PREDICTED:_probable_dolichyl_pyrophosphate_Ooceraea_biroi	10	2.05	0.8	1.07	0.78	0.9	0.86	0.24	0.90	1.16		
XP_002429694.1_dolichyl_glycosyltransferase_putative_Pediculus_humanus_corporis	11	1.88	1	1.03	0.91	1.09	1.67	1.07	1.26	0.98	1.03	
