# Supplementary material for: Pre-Columbian zoonotic enteric parasites: An insight into Puerto Rican indigenous culture diets and life styles
Source: PLoS One. 2020 Jan 30;15(1):e0227810. doi: 10.1371/journal.pone.0227810 (PMC6992007; doi:10.1371/journal.pone.0227810)
Supplement: S14 Table — (PDF) [file pone.0227810.s027.pdf]

S14 Table. BlastN homologous results of M01522:132:000000000-A4LNU:1:1111:24132:22042.1

	Specie ID	Max Score	Total Score	Query Cover	E-Value	Identification	Accession
M01522:132:000000000-A4LNU:1:1111:24132:22042.1	Schistosoma mansoni dolichyl glycosyltransferase partial mRNA	111	111	0.59	1E-20	0.77	XM_018795619.1
	Schistosoma mattheei genome assembly S_mattheei_Denwood	111	111	0.61	1E-20	0.77	LM158041.1
	Schistosoma rodhaini genome assembly S_rodhaini_Burundi	111	111	0.59	1E-20	0.77	LL957621.1
	Schistosoma mansoni strain Puerto Rico chromosome 2	111	111	0.59	1E-20	0.77	HE601625.1
	Schistosoma curassoni genome assembly S_curassoni_Dakar	107	107	0.61	1E-19	0.76	LM076444.1
	Schistosoma haematobium Dolichyl pyrophosphate	104	104	0.61	2E-18	0.75	XM_012945398.1
	Schistosoma japonicum isolate Anhui full length mRNA clone SJFCE287	102	102	0.59	6E-18	0.75	FN318736.1
	Schistosoma japonicum isolate Anhui full length mRNA clone SJFCE287	98.7	98.7	0.59	8E-17	0.75	FN318735.1
	Schistosoma japonicum SJCHGC03673 protein mRNA	96.9	96.9	0.59	3E-16	0.74	AY810735.1
	Trichobilharzia regenti genome assembly T_regenti_v1_0_4	93.3	93.3	0.57	3E-15	0.75	LL014407.1
