# Supplementary material for: Pre-Columbian zoonotic enteric parasites: An insight into Puerto Rican indigenous culture diets and life styles
Source: PLoS One. 2020 Jan 30;15(1):e0227810. doi: 10.1371/journal.pone.0227810 (PMC6992007; doi:10.1371/journal.pone.0227810)
Supplement: S15 Table — The number of base substitutions per site from between sequences are shown. Analyses were conducted using the Tamura 3-parameter model. (PDF) [file pone.0227810.s028.pdf]

S15 Table. Estimates of Evolutionary Divergence between Sequences (BlastN homology search of M01522:132:000000000-A4LNU:1:1111:24132:22042.1). The number of base substitutions per site from between sequences are shown. Analyses were conducted using the Tamura 3-parameter model.

		1	2	3	4	5	6	7	8	9	10	11
M01522:132:000000000-A4LNU:1:1111:24132:22042.1	1											
XM_018795619.1_Schistosoma_mansoni_dolichyl_glycosyltransferase_partial_mRNA	2	0.75										
LM158041.1_Schistosoma_mattheei_genome_assembly	3	0.86	0.06									
LL957621.1_Schistosoma_rodhaini_genome_assembly	4	0.33	0.59	0.65								
HE601625.1_Schistosoma_mansoni_strain_Puerto_Rico_chromosome_2	5	0.34	0.64	0.71	0.03							
LM076444.1_Schistosoma_curassoni_genome_assembly	6	0.81	0.05	0.01	0.61	0.68						
XM_012945398.1_Schistosoma_haematobium_Dolichyl_pyrophosphate	7	0.73	0.07	0.03	0.59	0.64	0.02					
FN318736.1_Schistosoma_japonicum_isolate_Anhui_full_length_mRNA_clone_SJFCE2873.003	8	0.83	0.15	0.18	0.60	0.66	0.16	0.18				
FN318735.1_Schistosoma_japonicum_isolate_Anhui_full_length_mRNA_clone_SJFCE2873.002	9	0.85	0.16	0.19	0.58	0.67	0.17	0.19	0.01			
AY810735.1_Schistosoma_japonicum_SJCHGC03673_protein_mRNA_partial_cds	10	0.85	0.16	0.19	0.58	0.67	0.17	0.19	0.01	0		
LL014407.1_Trichobilharzia_regenti_genome_assembly	11	0.35	0.72	0.84	0.27	0.25	0.80	0.76	0.79	0.80	0.80	
