# Supplementary material for: Pre-Columbian zoonotic enteric parasites: An insight into Puerto Rican indigenous culture diets and life styles
Source: PLoS One. 2020 Jan 30;15(1):e0227810. doi: 10.1371/journal.pone.0227810 (PMC6992007; doi:10.1371/journal.pone.0227810)
Supplement: S16 Table — (PDF) [file pone.0227810.s029.pdf]

S16 Table. BlastX homologous results of M01522:132:000000000-A4LNU:1:1102:17521:21100.1

	Specie ID	Max Score	Total Score	Query Cover	E-Value	Identification	Accession
M01522:132:000000000-A4LNU:1:1102:17521:21100.1	hypothetical protein NECAME_18601 [Necator americanus]	96.3	96.3	0.98	1E-21	0.56	XP_013295164.1
	hypothetical protein SI65_10228 [Aspergillus cristatus]	95.9	95.9	0.92	4E-21	0.57	ODM14393.1
	hypothetical protein SI65_07298 [Aspergillus cristatus]	94.7	94.7	0.98	1E-20	0.52	ODM17623.1
	hypothetical protein SI65_01726 [Aspergillus cristatus]	93.6	93.6	0.98	3E-20	0.52	ODM24136.1
	hypothetical protein SI65_09185 [Aspergillus cristatus]	93.6	93.6	0.98	3E-20	0.52	ODM15244.1
	ribonuclease H-like protein [Calocera cornea HHB12733]	86.7	86.7	0.98	5E-18	0.51	KZT62760.1
	protein of unknown function [Taphrina deformans PYCC 5710]	86.3	86.3	0.98	9E-18	0.49	CCG84601.2
	hypothetical protein B5P42_31445 [Bacillus sp. SRB_331]	80.5	80.5	0.96	2E-17	0.51	RAN68448.1
	hypothetical protein [Parasitella parasitica]	84	84	0.98	6E-17	0.49	CEP07157.1
	Transposon Tf2-12 polyprotein [Trametes pubescens]	84	84	0.98	7E-17	0.48	OJT11278.1
	hypothetical protein [Parasitella parasitica]	84	84	0.98	7E-17	0.49	CEP09716.1
	hypothetical protein [Absidia glauca]	82.8	82.8	0.98	2E-16	0.52	SAL96256.1
	retrovirus polyprotein, putative [Talaromyces marneffei ATCC 18224]	82	82	0.98	3E-16	0.46	XP_002145184.1
	hypothetical protein [Absidia glauca]	81.3	81.3	0.98	5E-16	0.54	SAM05196.1
	Transposon Tf2-6 polyprotein [Talaromyces marneffei PM1]	81.3	81.3	0.98	6E-16	0.46	KFX51368.1
	Retrotransposable element Tf2 155 kDa protein type 1 [Ceratocystis platani]	80.9	80.9	0.98	7E-16	0.48	KKF92157.1
	probable polyprotein-rice blast fungus magnaporthe gypsy retrotransposon	80.9	80.9	0.98	9E-16	0.45	T18348
	Transposon Tf2-12 polyprotein [Trametes pubescens]	80.1	80.1	0.98	1E-15	0.48	OJT06055.1
	retrotransposable element Tf2 155 kDa protein type 1-like [Mucor ambiguus]	80.1	80.1	0.98	1E-15	0.48	GAN03854.1
	hypothetical protein SCLCIDRAFT_28376 [Scleroderma citrinum Foug A]	79.3	79.3	0.98	2E-15	0.46	KIM58096.1
