# Supplementary material for: Pre-Columbian zoonotic enteric parasites: An insight into Puerto Rican indigenous culture diets and life styles
Source: PLoS One. 2020 Jan 30;15(1):e0227810. doi: 10.1371/journal.pone.0227810 (PMC6992007; doi:10.1371/journal.pone.0227810)
Supplement: S17 Table — The number of amino acid substitutions per site from between sequences are shown. Analyses were conducted using the JTT matrix-based model. (PDF) [file pone.0227810.s030.pdf]

S17 Table. Estimates of Evolutionary Divergence between Sequences (BlastX homology search of M01522:132:000000000-A4LNU:1:1102:17521:21100.1). The number of amino acid substitutions per site from between sequences are shown. Analyses were conducted using the JTT matrix-based model.

<table>
<tr><th></th><th></th><th>1</th><th>2</th><th>3</th><th>4</th><th>5</th><th>6</th><th>7</th><th>8</th><th>9</th><th>10</th><th>11</th><th>12</th><th>13</th><th>14</th><th>15</th><th>16</th><th>17</th><th>18</th><th>19</th><th>20</th><th>21</th></tr>
<tr><td>M01522:132:000000000-A4LNU:1:1102:17521:21100.1_frame+3</td><td>1</td><td></td><td></td><td></td><td></td><td></td><td></td><td></td><td></td><td></td><td></td><td></td><td></td><td></td><td></td><td></td><td></td><td></td><td></td><td></td><td></td><td></td></tr>
<tr><td>XP_013295164.1_hypothetical_protein_NECAME_18601_partial_Necator_americanus</td><td>2</td><td>0.75</td><td></td><td></td><td></td><td></td><td></td><td></td><td></td><td></td><td></td><td></td><td></td><td></td><td></td><td></td><td></td><td></td><td></td><td></td><td></td><td></td></tr>
<tr><td>ODM14393.1_hypothetical_protein_SI65_10228_Aspergillus_cristatus</td><td>3</td><td>0.79</td><td>0.81</td><td></td><td></td><td></td><td></td><td></td><td></td><td></td><td></td><td></td><td></td><td></td><td></td><td></td><td></td><td></td><td></td><td></td><td></td><td></td></tr>
<tr><td>ODM17623.1_hypothetical_protein_SI65_07298_Aspergillus_cristatus</td><td>4</td><td>0.87</td><td>0.77</td><td>0.12</td><td></td><td></td><td></td><td></td><td></td><td></td><td></td><td></td><td></td><td></td><td></td><td></td><td></td><td></td><td></td><td></td><td></td><td></td></tr>
<tr><td>ODM24136.1_hypothetical_protein_SI65_01726_Aspergillus_cristatus</td><td>5</td><td>0.87</td><td>0.77</td><td>0.12</td><td>0</td><td></td><td></td><td></td><td></td><td></td><td></td><td></td><td></td><td></td><td></td><td></td><td></td><td></td><td></td><td></td><td></td><td></td></tr>
<tr><td>ODM15244.1_hypothetical_protein_SI65_09185_Aspergillus_cristatus</td><td>6</td><td>0.87</td><td>0.77</td><td>0.12</td><td>0</td><td>0</td><td></td><td></td><td></td><td></td><td></td><td></td><td></td><td></td><td></td><td></td><td></td><td></td><td></td><td></td><td></td><td></td></tr>
<tr><td>KZT62760.1_ribonuclease_H-like_protein_partial_Calocera_cornea_HHB12733</td><td>7</td><td>1.06</td><td>0.93</td><td>1.15</td><td>1.02</td><td>1.02</td><td>1.02</td><td></td><td></td><td></td><td></td><td></td><td></td><td></td><td></td><td></td><td></td><td></td><td></td><td></td><td></td><td></td></tr>
<tr><td>CCG84601.2_protein_of_unknown_function_Taphrina_deformans_PYCC_5710</td><td>8</td><td>1</td><td>0.84</td><td>1.12</td><td>0.97</td><td>0.97</td><td>0.97</td><td>0.63</td><td></td><td></td><td></td><td></td><td></td><td></td><td></td><td></td><td></td><td></td><td></td><td></td><td></td><td></td></tr>
<tr><td>RAN68448.1_hypothetical_protein_B5P42_31445_partial_Bacillus_sp._SRB_331</td><td>9</td><td>1.17</td><td>0.97</td><td>1.15</td><td>0.98</td><td>0.98</td><td>0.98</td><td>0.47</td><td>0.64</td><td></td><td></td><td></td><td></td><td></td><td></td><td></td><td></td><td></td><td></td><td></td><td></td><td></td></tr>
<tr><td>CEP07157.1_hypothetical_protein_Parasitella_parasitica</td><td>10</td><td>1.09</td><td>0.72</td><td>0.89</td><td>0.76</td><td>0.76</td><td>0.76</td><td>0.6</td><td>0.51</td><td>0.45</td><td></td><td></td><td></td><td></td><td></td><td></td><td></td><td></td><td></td><td></td><td></td><td></td></tr>
<tr><td>OJT11278.1_Transposon_Tf2-12_polyprotein_Trametes_pubescens</td><td>11</td><td>1.24</td><td>1.03</td><td>1.32</td><td>1.25</td><td>1.25</td><td>1.25</td><td>0.56</td><td>0.72</td><td>0.83</td><td>0.75</td><td></td><td></td><td></td><td></td><td></td><td></td><td></td><td></td><td></td><td></td><td></td></tr>
<tr><td>CEP09716.1_hypothetical_protein_Parasitella_parasitica</td><td>12</td><td>1.09</td><td>0.72</td><td>0.89</td><td>0.76</td><td>0.76</td><td>0.76</td><td>0.6</td><td>0.51</td><td>0.45</td><td>0</td><td>0.75</td><td></td><td></td><td></td><td></td><td></td><td></td><td></td><td></td><td></td><td></td></tr>
<tr><td>SAL96256.1_hypothetical_protein_Absidia_glauca</td><td>13</td><td>0.93</td><td>0.79</td><td>1.07</td><td>0.91</td><td>0.91</td><td>0.91</td><td>0.44</td><td>0.69</td><td>0.61</td><td>0.53</td><td>0.79</td><td>0.53</td><td></td><td></td><td></td><td></td><td></td><td></td><td></td><td></td><td></td></tr>
<tr><td>XP_002145184.1_retrovirus_polyprotein_putative_Talaromyces_marneffei_ATCC_182</td><td>14</td><td>1.18</td><td>1.17</td><td>0.86</td><td>0.81</td><td>0.81</td><td>0.81</td><td>0.79</td><td>1.03</td><td>0.99</td><td>0.85</td><td>1.06</td><td>0.85</td><td>1.01</td><td></td><td></td><td></td><td></td><td></td><td></td><td></td><td></td></tr>
<tr><td>SAM05196.1_hypothetical_protein_Absidia_glauca</td><td>15</td><td>0.97</td><td>0.83</td><td>1.07</td><td>0.9</td><td>0.9</td><td>0.9</td><td>0.38</td><td>0.74</td><td>0.66</td><td>0.52</td><td>0.79</td><td>0.52</td><td>0.11</td><td>0.97</td><td></td><td></td><td></td><td></td><td></td><td></td><td></td></tr>
<tr><td>KFX51368.1_Transposon_Tf2-6_polyprotein_Talaromyces_marneffei_PM1</td><td>16</td><td>1.18</td><td>1.17</td><td>0.86</td><td>0.81</td><td>0.81</td><td>0.81</td><td>0.79</td><td>1.03</td><td>0.99</td><td>0.85</td><td>1.06</td><td>0.85</td><td>1.01</td><td>0</td><td>0.97</td><td></td><td></td><td></td><td></td><td></td><td></td></tr>
<tr><td>KKF92157.1_Retrotransposable_element_protein_type1_Ceratocystis_platani</td><td>17</td><td>1.17</td><td>0.8</td><td>0.42</td><td>0.38</td><td>0.38</td><td>0.38</td><td>1.05</td><td>1.16</td><td>1.11</td><td>0.98</td><td>1.29</td><td>0.98</td><td>1.11</td><td>0.77</td><td>1.02</td><td>0.77</td><td></td><td></td><td></td><td></td><td></td></tr>
<tr><td>pir||T18348_probable_polyprotein-_rice_blast_fungus_magnaporthe_gypsy_retrotra</td><td>18</td><td>1.14</td><td>0.9</td><td>0.35</td><td>0.26</td><td>0.26</td><td>0.26</td><td>0.93</td><td>1.09</td><td>1.04</td><td>0.91</td><td>1.32</td><td>0.91</td><td>0.94</td><td>0.79</td><td>0.92</td><td>0.79</td><td>0.31</td><td></td><td></td><td></td><td></td></tr>
<tr><td>OJT06055.1_Transposon_Tf2-12_polyprotein_Trametes_pubescens</td><td>19</td><td>1.38</td><td>1.16</td><td>1.35</td><td>1.18</td><td>1.18</td><td>1.18</td><td>0.5</td><td>0.78</td><td>0.82</td><td>0.7</td><td>0.29</td><td>0.7</td><td>0.65</td><td>1.15</td><td>0.65</td><td>1.15</td><td>1.19</td><td>1.24</td><td></td><td></td><td></td></tr>
<tr><td>GAN03854.1_retrotransposable_element_Tf2_155_kDa_protein_type_1-like_Mucor_</td><td>20</td><td>1.1</td><td>0.7</td><td>0.93</td><td>0.78</td><td>0.78</td><td>0.78</td><td>0.57</td><td>0.46</td><td>0.58</td><td>0.14</td><td>0.68</td><td>0.14</td><td>0.45</td><td>0.9</td><td>0.44</td><td>0.9</td><td>0.99</td><td>0.92</td><td>0.6</td><td></td><td></td></tr>
<tr><td>KIM58096.1_hypothetical_protein_SCLCIDRAFT_28376_Scleroderma_citrinum_Foug_</td><td>21</td><td>1.34</td><td>0.99</td><td>1.3</td><td>1.32</td><td>1.32</td><td>1.32</td><td>0.89</td><td>0.85</td><td>1.02</td><td>1.15</td><td>1.32</td><td>1.15</td><td>0.97</td><td>1.54</td><td>1.16</td><td>1.54</td><td>1.58</td><td>1.42</td><td>1.19</td><td>1.12</td><td></td></tr>
</table>
