# Supplementary material for: Pre-Columbian zoonotic enteric parasites: An insight into Puerto Rican indigenous culture diets and life styles
Source: PLoS One. 2020 Jan 30;15(1):e0227810. doi: 10.1371/journal.pone.0227810 (PMC6992007; doi:10.1371/journal.pone.0227810)
Supplement: S18 Table — (PDF) [file pone.0227810.s031.pdf]

S18 Table. BlastX homologous results of M01522:132:000000000-A4LNU:1:2109:13140:20960.1.

| | Specie ID | Max Score | Total Score | Query Cover | E-Value | Identification | Accession |
|---|---|---|---|---|---|---|---|
| M01522:132:000000000-A4LNU:1:2109:13140:20960.1 | conserved hypothetical protein [Perkinsus marinus ATCC 50983] | 82.4 | 82.4 | 0.86 | 3E-18 | 0.5 | XP_002769198.1 |
| | hypothetical protein Pmar_PMAR024486 [Perkinsus marinus ATCC 50983] | 81.3 | 81.3 | 0.84 | 5e-16 | 0.51 | XP_002767291.1 |
| | hypothetical protein AN2428.2 [Aspergillus nidulans FGSC A4] | 76.3 | 76.3 | 0.59 | 3e-14 | 0.57 | XP_660032.1 |
| | Zinc finger, RING/FYVE/PHD-type [Umbilicaria pustulata] | 75.9 | 75.9 | 0.59 | 4e-14 | 0.54 | SLM39322.1 |
| | TPA: SH3 domain protein (AFU_orthologue; AFUA_2G13880) [Aspergillus nidulans FGSC A4] | 75.1 | 75.1 | 0.65 | 7e-14 | 0.53 | CBF86833.1 |
| | hypothetical protein PDIP_78730 [Penicillium digitatum Pd1] | 74.3 | 74.3 | 0.59 | 8e-14 | 0.6 | XP_014532030.1 |
| | hypothetical protein AOQ84DRAFT_435406 [Glonium stellatum] | 74.7 | 74.7 | 0.77 | 1e-13 | 0.47 | OCL14819.1 |
| | hypothetical protein PENANT_c019G07953 [Penicillium antarcticum] | 74.3 | 74.3 | 0.63 | 1e-13 | 0.59 | OQD82820.1 |
| | hypothetical protein K432DRAFT_348117 [Lepidopterella palustris CBS 459.81] | 74.3 | 74.3 | 0.74 | 1e-13 | 0.49 | OCK82894.1 |
| | hypothetical protein BAUCODRAFT_392037 [Baudoinia panamericana UAMH 10762] | 74.3 | 74.3 | 0.66 | 1e-13 | 0.48 | XP_007674127.1 |
