# Supplementary material for: Pre-Columbian zoonotic enteric parasites: An insight into Puerto Rican indigenous culture diets and life styles
Source: PLoS One. 2020 Jan 30;15(1):e0227810. doi: 10.1371/journal.pone.0227810 (PMC6992007; doi:10.1371/journal.pone.0227810)
Supplement: S19 Table — The number of amino acid substitutions per site from between sequences are shown. Analyses were conducted using the Dayhoff matrix based model. (PDF) [file pone.0227810.s032.pdf]

S19 Table. Estimates of Evolutionary Divergence between Sequences (BlastX homology search of M01522:132:000000000-A4LNU:1:2109:13140:20960.1). The number of amino acid substitutions per site from between sequences are shown. Analyses were conducted using the Dayhoff matrix based model.

		1	2	3	4	5	6	7	8	9	10	11
M01522:132:000000000-A4LNU:1:2109:13140:20960.1	1											
XP_002769198.1_conserved_hypothetical_protein_Perkinsus_marinus_ATCC_50983	2	0.72										
XP_002767291.1_hypothetical_protein_Pmar_PMAR024486_Perkinsus_marinus_ATCC_50983	3	0.72	0									
XP_660032.1_hypothetical_protein_AN2428.2_Aspergillus_nidulans_FGSC_A4	4	0.45	0.96	0.96								
SLM39322.1_Zinc_finger_RING/FYVE/PHD-type_Umbilicaria_pustulata	5	0.54	0.97	0.97	0.18							
CBF86833.1_TPA:_SH3_domain_protein_(AFU_orthologue_AFUA_2G13880)_Aspergillus_nidulans_FGSC_A4	6	0.45	0.96	0.96	0	0.18						
XP_014532030.1_hypothetical_protein_PDIP_78730_Penicillium_digitatum_Pd1	7	0.52	1.01	1.01	0.21	0.24	0.21					
OCL14819.1_hypothetical_protein_AOQ84DRAFT_435406_Glonium_stellatum	8	0.53	0.89	0.89	0.26	0.1	0.26	0.32				
OQD82820.1_hypothetical_protein_PENANT_c019G07953_Penicillium_antarcticum	9	0.53	1.03	1.03	0.18	0.24	0.18	0.07	0.28			
OCK82894.1_hypothetical_protein_K432DRAFT_348117_Lepidopterella_palustris_CBS_459.81	10	0.53	0.89	0.89	0.26	0.12	0.26	0.32	0.02	0.28		
XP_007674127.1_hypothetical_protein_BAUCODRAFT_392037_Baudoinia_panamericana_UAMH_10762	11	0.61	1.01	1.01	0.25	0.25	0.25	0.34	0.23	0.31	0.25	
