# Supplementary material for: Pre-Columbian zoonotic enteric parasites: An insight into Puerto Rican indigenous culture diets and life styles
Source: PLoS One. 2020 Jan 30;15(1):e0227810. doi: 10.1371/journal.pone.0227810 (PMC6992007; doi:10.1371/journal.pone.0227810)
Supplement: S20 Table — (PDF) [file pone.0227810.s033.pdf]

S20 Table. BlastX homologous results of M01522:132:000000000-A4LNU:1:2108:6882:8618.

	Specie ID	Max Score	Total Score	Query Cover	E-Value	Identification	Accession
M01522:132:000000000-A4LNU:1:2108:6882:8618	branched-chain alpha-keto acid dehydrogenase E1 component beta chain[Eimeria necatrix]	139	139	0.98	2E-38	0.72	XP_013437400.1
	unnamed protein product [Vitrella brassicaformis CCMP3155]	139	139	0.98	3e-38	0.74	CEM30924.1
	RabGAP/TBC domain-containing protein [Heterostelium album PN500]	140	140	0.98	1e-36	0.73	XP_020429359.1
	3-methyl-2-oxobutanoate dehydrogenase [Tieghemostelium lacteum]	135	135	0.98	2e-36	0.73	KYQ91940.1
	branched-chain alpha-KETO ACID decarboxylase E1 beta subunit [Acanthamoeba castellanii]	135	135	0.98	2e-36	0.73	XP_004368155.1
	branched-chain alpha-keto acid dehydrogenase E1 component beta chain [Eimeria tenella]	135	135	0.98	3e-36	0.72	XP_013228676.1
	Thiamin diphosphate-binding protein [Daedalea quercina L-15889]	135	135	0.98	5e-36	0.74	KZT71260.1
	branched-chain alpha-keto acid dehydrogenase E1 component beta chain [Eimeria brunetti]	133	133	0.98	6e-36	0.71	CDJ50268.1
	pyruvate dehydrogenase [Sanghuangporus baumii]	135	135	0.98	6e-36	0.7	OCB86717.1
	branched-chain alpha-keto acid dehydrogenase E1 component beta chain [Eimeria mitis]	132	132	0.98	1e-35	0.71	XP_013355591.1
