# Supplementary material for: Pre-Columbian zoonotic enteric parasites: An insight into Puerto Rican indigenous culture diets and life styles
Source: PLoS One. 2020 Jan 30;15(1):e0227810. doi: 10.1371/journal.pone.0227810 (PMC6992007; doi:10.1371/journal.pone.0227810)
Supplement: S21 Table — The number of amino acid substitutions per site from between sequences are shown. Analyses were conducted using the JTT matrix-based model. (PDF) [file pone.0227810.s034.pdf]

S21 Table. Estimates of Evolutionary Divergence between Sequences (BlastX homology search of M01522:132:000000000-A4LNU:1:2108:6882:8618). The number of amino acid substitutions per site from between sequences are shown. Analyses were conducted using the JTT matrix-based model.

		1	2	3	4	5	6	7	8	9	10	11
M01522:132:000000000-A4LNU:1:2108:6882:8618	1											
XP_013437400.1_branched-chain_alpha-keto_acid_dehydrogenase_E1_component_beta_chain_putative_Eimeria_necatrix	2	0.18										
CEM30924.1_unnamed_protein_product_Vitrella_brassicaformis_CCMP3155	3	0.14	0.14									
XP_020429359.1_RabGAP/TBC_domain-containing_protein_Heterostelium_album_PN500	4	0.18	0.27	0.26								
KYQ91940.1_3-methyl-2-oxobutanoate_dehydrogenase_Tieghemostelium_lacteum	5	0.17	0.27	0.27	0.06							
XP_004368155.1_branched-chain_alpha-KETO_ACID_decarboxylase_E1_beta_subunit_putative_Acanthamoeba_castellanii	6	0.19	0.36	0.21	0.25	0.27						
XP_013228676.1_branched-chain_alpha-keto_acid_dehydrogenase_E1_component_beta_chain_putative_Eimeria_tenella	7	0.18	0	0.14	0.27	0.27	0.36					
KZT71260.1_Thiamin_diphosphate-binding_protein_Daedalea_quercina_L-15889	8	0.24	0.34	0.27	0.33	0.39	0.35	0.34				
CDJ50268.1_branched-chain_alpha-keto_acid_dehydrogenase_E1_component_beta_chain_putative_Eimeria_brunetti	9	0.19	0.03	0.14	0.25	0.28	0.38	0.03	0.33			
OCB86717.1_pyruvate_dehydrogenase_Sanghuangporus_baumii	10	0.26	0.36	0.29	0.35	0.41	0.37	0.36	0.04	0.35		
XP_013355591.1_branched-chain_alpha-keto_acid_dehydrogenase_E1_component_beta_chain_putative_Eimeria_mitis	11	0.19	0.03	0.14	0.25	0.28	0.38	0.03	0.33	0	0.35	
