# Supplementary material for: Pre-Columbian zoonotic enteric parasites: An insight into Puerto Rican indigenous culture diets and life styles
Source: PLoS One. 2020 Jan 30;15(1):e0227810. doi: 10.1371/journal.pone.0227810 (PMC6992007; doi:10.1371/journal.pone.0227810)
Supplement: S22 Table — (PDF) [file pone.0227810.s035.pdf]

S22 Table. BlastN homologous results of M01522:132:000000000-A4LNU:1:2108:6882:8618.

	Specie ID	Max Score	Total Score	Query Cover	E-Value	Identification	Accession
M01522:132:000000000-A4LNU:1:2108:6882:8618	Eimeria mitis branched-chain alpha-keto acid dehydrogenase E1 component beta chain	152	152	0.7	4E-33	0.77	XM_013500137.1
	Aphanomyces euteiches cDNA	150	150	0.71	2E-32	0.77	CU355634.1
	Aphanomyces invadans 2-oxoisovalerate dehydrogenase subunit beta, mitochondrial mRNA	145	145	0.71	7E-31	0.76	XM_008875086.1
	Aphanomyces astaci 2-oxoisovalerate dehydrogenase subunit beta, mitochondrial mRNA	143	143	0.7	2E-30	0.76	XM_009831314.1
	Naegleria gruberi predicted protein, mRNA	141	141	0.71	8E-30	0.76	XM_002673181.1
	Lichtheimia ramosa strain JMRC FSU:6197 genome assembly, scaffold: SCAF2	140	140	0.73	3E-29	0.75	LK023324.1
	PREDICTED: Orussus abietinus 2-oxoisovalerate dehydrogenase subunit beta, mitochondrial (	138	138	0.7	1E-28	0.76	XM_012421320.2
	PREDICTED: Orussus abietinus 2-oxoisovalerate dehydrogenase subunit beta, mitochondrial (	138	138	0.7	1E-28	0.76	XM_012421319.2
	PREDICTED: Orussus abietinus 2-oxoisovalerate dehydrogenase subunit beta, mitochondrial (	138	138	0.7	1E-28	0.76	XM_012421318.2
	Batrachochytrium dendrobatidis JAM81 hypothetical protein (BATDEDRAFT_88045), partial	138	138	0.72	1E-28	0.76	XM_006678602.1
