# Supplementary material for: Pre-Columbian zoonotic enteric parasites: An insight into Puerto Rican indigenous culture diets and life styles
Source: PLoS One. 2020 Jan 30;15(1):e0227810. doi: 10.1371/journal.pone.0227810 (PMC6992007; doi:10.1371/journal.pone.0227810)
Supplement: S23 Table — The number of base substitutions per site from between sequences are shown. Analyses were conducted using the Kimura 2-parameter model. (PDF) [file pone.0227810.s036.pdf]

S23 Table. Estimates of Evolutionary Divergence between Sequences (BlastN homology search of M01522:132:000000000-A4LNU:1:2108:6882:8618). The number of base substitutions per site from between sequences are shown. Analyses were conducted using the Kimura 2-parameter model.

		1	2	3	4	5	6	7
**M01522:132:000000000-A4LNU:1:2108:6882:8618**	**1**							
**XM_013500137.1_Eimeria_mitis_branched-chain_alpha-keto_acid_dehydrogenase**	**2**	0.87						
**CU355634.1_Aphanomyces_euteiches_cDNA**	**3**	0.87	2.45					
**XM_008875086.1_Aphanomyces_invadans_2-oxoisovalerate_dehydrogenase_subunit_beta_mitochondrial_mRNA**	**4**	1.03	2.14	0.39				
**XM_009831314.1_Aphanomyces_astaci_2-oxoisovalerate_dehydrogenase_subunit_beta_mitochondrial_mRNA**	**5**	0.98	2.14	0.48	0.29			
**XM_002673181.1_Naegleria_gruberi_predicted_protein_mRNA**	**6**	0.95	1.65	1.16	2.45	2.12		
**LK023324.1_Lichtheimia_ramosa_strain_JMRC_FSU:6197_genome_assembly_scaffold:_SCAF2**	**7**	1.32	2.62	1.65	1.92	1.92	1.88	
