# Supplementary material for: Pre-Columbian zoonotic enteric parasites: An insight into Puerto Rican indigenous culture diets and life styles
Source: PLoS One. 2020 Jan 30;15(1):e0227810. doi: 10.1371/journal.pone.0227810 (PMC6992007; doi:10.1371/journal.pone.0227810)
Supplement: S24 Table — (PDF) [file pone.0227810.s037.pdf]

S24 Table. BlastX homologous results of M01522:132:000000000-A4LNU:1:2110:20683:15891.1

	Specie ID	Max Score	Total Score	Query Cover	E-Value	Identification	Accession
M01522:132:000000000-A4LNU:1:2110:20683:15891.1	expressed protein [Hymenolepis microstoma]	110	110	0.9	2e-29	0.93	CDS30781.1
	hypothetical transcript [Hymenolepis microstoma]	110	110	0.9	8e-29	0.93	CUU98466.1
	unknown [Schistosoma japonicum]	105	105	0.89	2e-28	0.91	AAX30301.1
	unknown [Picea sitchensis]	104	104	0.89	2e-28	0.93	ABR16542.1
	hypothetical protein L798_05095 [Zootermopsis nevadensis]	105	105	0.9	9e-28	0.89	KDR02726.1
	hypothetical protein LEMA_P113070.1 [Leptosphaeria maculans JN3]	102	102	0.79	1e-27	1	XP_003839867.1
	hypothetical protein SMU82_09667 [Streptococcus mutans SM6]	102	102	0.81	1e-27	0.98	EMC20828.1
	hypothetical protein COCSUDRAFT_19418 [Coccomyxa subellipsoidea C-169]	102	102	0.89	1e-27	0.91	XP_005644353.1
	hypothetical protein THAOC_21443 [Thalassiosira oceanica]	103	103	0.89	1e-27	0.91	EJK58441.1
	hypothetical protein CHLNCDRAFT_48520 [Chlorella variabilis]	102	102	0.89	2e-27	0.91	XP_005850831.1
