# Supplementary material for: Pre-Columbian zoonotic enteric parasites: An insight into Puerto Rican indigenous culture diets and life styles
Source: PLoS One. 2020 Jan 30;15(1):e0227810. doi: 10.1371/journal.pone.0227810 (PMC6992007; doi:10.1371/journal.pone.0227810)
Supplement: S25 Table — The number of amino acid substitutions per site from between sequences are shown. Analyses were conducted using the JTT matrix-based model. (PDF) [file pone.0227810.s038.pdf]

S25 Table. Estimates of Evolutionary Divergence between Sequences (BlastX homology search of M01522:132:000000000-A4LNU:1:2110:20683:15891.1). The number of amino acid substitutions per site from between sequences are shown. Analyses were conducted using the JTT matrix-based model.

		1	2	3	4	5	6	7	8	9	10	11
M01522:132:000000000-A4LNU:1:2110:20683:15891.1	1											
CDS30781.1_expressed_protein_Hymenolepis_microstoma	2	0.02										
CUU98466.1_hypothetical_transcript_Hymenolepis_microstoma	3	0.02	0									
AAX30301.1_unknown_Schistosoma_japonicum	4	0.09	0.08	0.08								
ABR16542.1_unknown_Picea_sitchensis	5	0.02	0	0	0.08							
KDR02726.1_hypothetical_protein_L798_05095_Zootermopsis_nevadensis	6	0.13	0.1	0.1	0.18	0.1						
XP_003839867.1_hypothetical_protein_LEMA_P113070.1_Leptosphaeria_maculans_JN3	7	0	0.02	0.02	0.09	0.02	0.13					
EMC20828.1_hypothetical_protein_SMU82_09667_partial_Streptococcus_mutans_SM6	8	0.02	0	0	0.08	0	0.1	0.02				
XP_005644353.1_hypothetical_protein_COCSUDRAFT_19418_Coccomyxa_subellipsoidea_C-169	9	0.07	0.05	0.05	0.11	0.05	0.08	0.07	0.05			
EJK58441.1_hypothetical_protein_THAOC_21443_Thalassiosira_oceanica	10	0.07	0.06	0.06	0.13	0.06	0.14	0.07	0.06	0.08		
XP_005850831.1_hypothetical_protein_CHLNCDRAFT_48520_Chlorella_variabilis	11	0.07	0.05	0.05	0.11	0.05	0.08	0.07	0.05	0	0.08	
