# Supplementary material for: Pre-Columbian zoonotic enteric parasites: An insight into Puerto Rican indigenous culture diets and life styles
Source: PLoS One. 2020 Jan 30;15(1):e0227810. doi: 10.1371/journal.pone.0227810 (PMC6992007; doi:10.1371/journal.pone.0227810)
Supplement: S26 Table — (PDF) [file pone.0227810.s039.pdf]

S26 Table. BlastX homologous results of M01522:132:000000000-A4LNU:1:2114:18798:18268

| | Specie ID | Max Score | Total Score | Query Cover | E-Value | Identification | Accession |
|---|---|---|---|---|---|---|---|
| M01522:132:000000000-A4LNU:1:2114:18798:18268 | putative DNA topoisomerase 2 [Toxoplasma gondii MAS] | 75.9 | 115 | 0.42 | 5E-17 | 0.64 | KFH17918.1 |
| | DNA topoisomerase 2, putative [Toxoplasma gondii ME49] | 75.9 | 115 | 0.42 | 6E-17 | 0.64 | XP_018635491.1 |
| | TPA: DNA topoisomerase II, putative [Toxoplasma gondii VEG] | 75.9 | 115 | 0.42 | 6E-17 | 0.64 | CEL77484.1 |
| | putative DNA topoisomerase 2 [Toxoplasma gondii ARI] | 75.9 | 115 | 0.42 | 6E-17 | 0.64 | KYF48073.1 |
| | putative DNA topoisomerase 2 [Toxoplasma gondii GT1] | 75.9 | 115 | 0.42 | 6E-17 | 0.64 | EPR61049.1 |
| | putative DNA topoisomerase 2 [Toxoplasma gondii] | 75.9 | 115 | 0.42 | 6E-17 | 0.64 | KFG44197.1 |
| | DNA topoisomerase 2, putative [Hammondia hammondi] | 75.5 | 114 | 0.42 | 8E-17 | 0.64 | XP_008885802.1 |
| | putative DNA topoisomerase 2 [Toxoplasma gondii RUB] | 75.1 | 114 | 0.42 | 8E-17 | 0.64 | KFG65986.1 |
| | putative DNA topoisomerase 2 [Toxoplasma gondii COUG] | 75.1 | 114 | 0.42 | 9E-17 | 0.64 | PIL98402.1 |
| | putative DNA topoisomerase 2 [Toxoplasma gondii FOU] | 75.1 | 114 | 0.42 | 9E-17 | 0.64 | KFG55993.1 |
| | putative DNA topoisomerase 2 [Toxoplasma gondii p89] | 75.9 | 114 | 0.42 | 9E-17 | 0.64 | KFG50294.1 |
| | putative DNA topoisomerase 2 [Toxoplasma gondii TgCatPRC2] | 75.5 | 113 | 0.42 | 1E-16 | 0.64 | KYK69862.1 |
| | putative DNA topoisomerase 2 [Toxoplasma gondii VAND] | 75.5 | 113 | 0.42 | 1E-16 | 0.64 | KFH09612.1 |
| | DNA topoisomerase II, partial [Nannizzia persicolor] | 76.3 | 156 | 0.58 | 2E-16 | 0.75 | BAD02204.1 |
