# Supplementary material for: Pre-Columbian zoonotic enteric parasites: An insight into Puerto Rican indigenous culture diets and life styles
Source: PLoS One. 2020 Jan 30;15(1):e0227810. doi: 10.1371/journal.pone.0227810 (PMC6992007; doi:10.1371/journal.pone.0227810)
Supplement: S27 Table — The number of amino acid substitutions per site from between sequences are shown. Analyses were conducted using the JTT matrix-based model. (PDF) [file pone.0227810.s040.pdf]

S27 Table. Estimates of Evolutionary Divergence between Sequences (BlastX homology search of M01522:132:000000000-A4LNU:1:2114:18798:18268). The number of amino acid substitutions per site from between sequences are shown. Analyses were conducted using the JTT matrix-based model.

		1	2	3	4	5	6	7	8	9	10	11	12	13	14	15	16
BAD02204.1_DNA_topoisomerase_II_partial_Nannizzia_persicolor	1																
CEL77484.1_TPA:_DNA_topoisomerase_II_putative_Toxoplasma_gondii_VEG	2	0.52															
EPR61049.1_putative_DNA_topoisomerase_2_Toxoplasma_gondii_GT1	3	0.52	0														
KFG44197.1_putative_DNA_topoisomerase_2_Toxoplasma_gondii	4	0.52	0	0													
KFG50294.1_putative_DNA_topoisomerase_2_Toxoplasma_gondii_p89	5	0.52	0	0	0												
KFG55993.1_putative_DNA_topoisomerase_2_partial_Toxoplasma_gondii_FOU	6	0.52	0	0	0	0											
KFG65986.1_putative_DNA_topoisomerase_2_partial_Toxoplasma_gondii_RUB	7	0.52	0	0	0	0	0										
KFH09612.1_putative_DNA_topoisomerase_2_partial_Toxoplasma_gondii_VANI	8	0.52	0	0	0	0	0	0									
KFH17918.1_putative_DNA_topoisomerase_2_Toxoplasma_gondii_MAS	9	0.52	0	0	0	0	0	0	0								
KYF48073.1_putative_DNA_topoisomerase_2_Toxoplasma_gondii_ARI	10	0.52	0	0	0	0	0	0	0	0							
KYK69862.1_putative_DNA_topoisomerase_2_Toxoplasma_gondii_TgCatPRC2	11	0.52	0	0	0	0	0	0	0	0	0						
M01522:132:000000000-A4LNU:1:2114:18798:18268	12	0.33	0.45	0.45	0.45	0.45	0.45	0.45	0.45	0.45	0.45	0.45					
PIL98402.1_putative_DNA_topoisomerase_2_partial_Toxoplasma_gondii_COUG	13	0.52	0	0	0	0	0	0	0	0	0	0	0.45				
XP_003885185.1_hypothetical_protein_NCLIV_055820_Neospora_caninum_Liver	14	0.52	0	0	0	0	0	0	0	0	0	0	0.45	0			
XP_008885802.1_DNA_topoisomerase_2_putative_Hammondia_hammondi	15	0.52	0	0	0	0	0	0	0	0	0	0	0.45	0	0		
XP_018635491.1_DNA_topoisomerase_2_putative_Toxoplasma_gondii_ME49	16	0.52	0	0	0	0	0	0	0	0	0	0	0.45	0	0	0	
